# Analysis of aging effects on the mechanical and vibration properties of quasi-isotropic basalt fiber-reinforced polymer composites

**DOI:** 10.1038/s41598-024-77374-x

**Published:** 2024-11-05

**Authors:** B Namrata, Yogeesha Pai, Vishnu G Nair, Navya Thirumaleshwar Hegde, Deepthi G Pai

**Affiliations:** 1 Department of Aeronautical and Automobile Engineering, Manipal Institute of Technology, Manipal Academy of Higher Education (MAHE), Manipal, 576 104 Karnataka India; 2 Department of Computer Science and Engineering, Manipal Institute of Technology, Manipal Academy of Higher Education (MAHE), Manipal, 576 104 Karnataka India

**Keywords:** Basalt composite, Quasi-isotropic orientation, Moisture absorption, Damping properties, Mechanical performance, Sound transmission loss, Engineering, Materials science

## Abstract

Eco-friendly natural fiber composites, such as basalt fiber composites, are gaining traction in material science but remain vulnerable to environmental degradation. This study investigates the mechanical and vibrational properties of quasi-isotropic basalt fiber composites subjected to aging in two different environments: ambient (30 ºC) and subzero (-10 ºC), both in distilled water until moisture saturation. Aged specimens absorbed 8.66% and 5.44% moisture in ambient and subzero conditions, respectively. Mechanical testing revealed significant strength reductions in tensile, flexural, impact, and short beam shear tests, with ambient-aged specimens showing the largest decline (up to 31.7% in flexural strength). Vibrational analysis showed reduced natural frequencies, particularly under ambient conditions (27.27%). Sound absorption tests showed that pristine specimens had the highest transmission loss, while moisture-rich ambient-aged specimens had the lowest. SEM analysis confirmed surface degradation, with fiber pull-out and matrix debonding contributing to property loss. This research provides valuable insights into the environmental limitations of basalt fiber composites, emphasizing the need for enhanced durability in eco-friendly materials.

##  Introduction

Composite materials have become a cornerstone of modern engineering and manufacturing, offering unparalleled advantages over traditional materials like metals and ceramics^1,2^. Fiber reinforced polymer (FRP) composites are widely used in aerospace, automotive, marine, and civil engineering due to their excellent strength-to-weight ratio, corrosion resistance, and design flexibility. Synthetic fibers, which have traditionally been the reinforcement of choice due to their high strength, durability, and cost-effectiveness, are increasingly being replaced by natural fibers^3,4^. Natural fibers, classified into plant, animal, and mineral categories, have become increasingly popular due to their eco-friendly and sustainable nature. Plant fibers, rich in cellulose, are derived from various parts of plants such as stems (e.g., flax, hemp, jute), leaves (e.g., sisal, abaca), seeds (e.g., cotton, kapok), fruits (e.g., coconut coir), and roots^5^. These fibers are widely used in industries for textiles, packaging, and composite materials. Animal fibers like wool, silk, and alpaca fiber, known for their warmth, insulation, and softness, are essential in the textile industry, while mineral fibers like basalt and asbestos (now avoided due to health risks) offer fire-resistant properties and are used in construction^6^. The demand for natural fibers continues to grow, especially in applications like automotive composites, where their lightweight, renewable, and biodegradable characteristics make them ideal for sustainable development^7,8^.

Basalt is one such natural material that has gained prominence as a natural alternative to synthetic fibers in polymer composites. Basalt fibers are increasingly used across various industries, including construction for reinforcing concrete, automotive for lightweight components, and aerospace for structural parts due to their lightweight and heat-resistant properties. Their corrosion resistance makes them ideal for marine applications, while their high melting point allows for use in fireproof materials and protective gear. Additionally, basalt fibers enhance the performance of sports equipment, such as skis and tennis rackets, by providing strength and flexibility^9–11^. Basalt fibers are manufactured through a sophisticated process that begins with melting basalt rock at elevated temperatures ranging from 1400 to 1700 °C^12^. The molten rock is then extruded through fine nozzles to create continuous fibers. These fibers are subsequently treated with sizing agents to enhance their bonding with polymer matrices in composite materials^13^. Basalt fibers are not only environmentally sustainable but also possess impressive mechanical characteristics, such as high tensile strength, thermal stability, and resistance to chemical degradation^14,15^. Basalt fibers have several potential applications in the aerospace sector since they have properties like E-glass fibers. Research studies have shown that they can replace E-glass fibers due to environmental benefits, manufacturing efficiencies, and cost considerations^16,17^. In the aerospace and automotive sectors, composite components need to exhibit strength in all directions to effectively handle loads and stresses that can be applied randomly or in multiple directions^18,19^. Ideally, a material with isotropic properties would be best suited for this requirement. However, because composite materials inherently possess directional properties due to the alignment of their embedded fibers, achieving true isotropy is not possible. As a result, the development and study of quasi-isotropic laminates become essential to approximate the performance characteristics of isotropic materials^20,21^.

Polymer composite structures when employed in outdoor applications are often subjected to a range of hygrothermal conditions, which can significantly degrade their mechanical properties^22–24^. Common forms of damage in fiber-reinforced polymers (FRPs) due to aging include micro matrix cracking, fiber-matrix interface debonding, and delamination, which can ultimately lead to structural failure^25,26^. Moisture ingress in FRPs is known to affect the matrix polymer, reinforced fibers, and the interfaces between laminae^22^. Since the matrix resin is an organic compound, prolonged exposure to moisture can induce both physical and chemical changes, resulting in a deterioration in mechanical properties^28,29^. Physical deterioration often manifests as plasticization and swelling of the polymer matrix, which can make the material more ductile and lower its glass transition temperature. Moisture diffusion can also cause hydrolysis of the polymeric matrix, degrading the fiber-matrix interface^30–32^.

Table 1 provides an overview of available literatures examining the aging of basalt fibers or basalt composites in various aging conditions. Most literatures on the aging of basalt composites have concentrated on exposure to saltwater and artificial seawater^33–36^. Wang et al^36^. investigated basalt fibers immersed in artificial seawater at elevated temperatures and observed an initial increase in mechanical characteristics, followed by a decline. Authors explained that the initial improvement was due to the smoothing of surface micro-cracks caused by corrosion of crack tips, while the subsequent decrease was attributed to the degradation of the glass network through corrosion. In contrast, Lu et al^35^. noted a significant decrease in mechanical properties when basalt fibers were immersed in artificial seawater at lower temperatures, but did not provide detailed insights into the degradation mechanisms. Research indicates that employing a polymer matrix can shield basalt fibers from degradation^35,37^, contributing to the improved durability as witnessed at the composite level in other investigations^33,34,38^. However, basalt fiber-reinforced composites generally exhibit greater sensitivity to water absorption compared to glass reinforced composites^35,38^. Whereas some researchers argue that the long-term behavior of both composite types is similar^33,38^, others have reported inferior resistance to seawater aging for basalt composites^39^, attributed to the formation of a corrosion layer that weakens the interfacial adhesion between the fiber and matrix.

**Table 1:** A compilation of literatures available on the effects of aging on basalt fibers or basalt laminates in various water-based conditions.

Published year and Reference	Sample type	Aging condition	Aging temperature	Aging duration	Findings
2009^40^	Basalt fibers	alkaline	20,40,60, 80 ℃	15 days	The mechanical characteristics are highly influenced by the chemistry of the solution.
2011^33^	Basalt laminate	artificial seawater	25 ℃	90 days	Tensile and bending strengths decreased with longer seawater exposure
2014^41^	Basalt fibers	alkaline	80 ℃	11 days	Formation of corrosion shell was observed
2015^42^	Basalt fibers + lamina	alkaline, acidic, salt water, tap water	25, 55 ℃	66 days	Basalt FRP composites showed better tensile properties than basalt fibers in all environments
2016^43^	Basalt laminate	alkaline	ambient	64 days	Zirconium dioxide reduces basalt fiber corrosion more effectively than titanium dioxide.
2016^37^	Basalt laminate	alkaline, acidic	100 ℃	3 h	Alkali environments can damage BF ropes and BFRP rods; protective coatings are necessary
2018^38^	Basalt laminate	natural seawater	4, 25, 40, 60 ℃	6 months 10 days	20% drop in interlaminar shear properties, and more significant reduction in flexural strength after saturation.
2018^44^	Basalt fibers	alkaline	25, 50, 70 ℃	3 days	Reduced tensile properties and increase in corrosion rate with temperature
2019^34^	Basalt laminate	artificial seawater	25 ℃	42 days	Tensile properties remain stable, but fatigue life is significantly impacted
2020^39^	Basalt laminate	alkaline, tap water, artificial sea water	20 ℃	6 months	Alkaline solutions severely weaken BFRP bars, but elastic modulus remains largely unchanged
2021^36^	Basalt fibers	artificial seawater	80, 85, 90 ℃	168 h	Tensile strength initially increases (18 h) but decreases afterward under the same conditions.
2022^35^	Basalt fibers + laminate	artificial seawater, distilled water	20,40, 60 ℃	3 months	Tensile and interlaminar shear strength decline more in distilled water than in artificial seawater.

Although extensive research has been conducted on basalt composites, most studies focus on seawater environments, with limited exploration of the effects of distilled water aging on their mechanical and vibrational properties. This study addresses a key research gap in the understanding of how eco-friendly basalt fiber composites perform under different environmental aging conditions, particularly in subzero and ambient environments. While natural fiber composites are recognized for their sustainability, there is limited research on their durability when exposed to prolonged moisture absorption and temperature variations. Most studies focus on mechanical properties but overlook vibrational behavior, which is crucial for structural applications. This research fills that gap by examining both mechanical and vibrational properties, providing a more complete picture of how aging affects these composites. The findings are significant for advancing the development of more durable, eco-friendly materials that can withstand real-world conditions, including moisture and temperature fluctuations.

Therefore, this investigation explores the quasi-isotropic basalt/epoxy composite, focusing on configurations suited for applications that require mechanical properties similar to those of thin and isotropic materials. The aging conditions of ambient 30 °C and subzero − 10 °C were chosen for the study to reflect real-world environmental exposures that basalt fiber composites may face. The 30 °C ambient temperature represents typical operating conditions in many regions, while the − 10 °C subzero temperature simulates harsher environments, such as cold climates or industrial applications where freezing conditions are common. These specific temperatures allow for a comprehensive assessment of how the material performs across a range of practical temperature extremes, helping to identify potential weaknesses in both every day and more severe environmental conditions.

The novel aspects of this research include its comprehensive analysis of both mechanical and vibrational properties of quasi-isotropic basalt fiber composites under ambient and subzero aging conditions. By focusing on both structural integrity and dynamic performance, the study provides valuable insights into the environmental durability of these composites. Its potential impact lies in guiding the development of more durable, eco-friendly materials for industries like construction, automotive, and aerospace, where temperature and moisture fluctuations are common. Hence, the aim of this study is to analyze the mechanical integrity and damping properties of basalt fabric-based quasi-isotropic composites subjected to various aging scenarios through experimental tests. These tests provide critical material characteristics essential for selecting appropriate materials for diverse applications.

##  Materials and procedures

### Materials

**Fig. 1:**
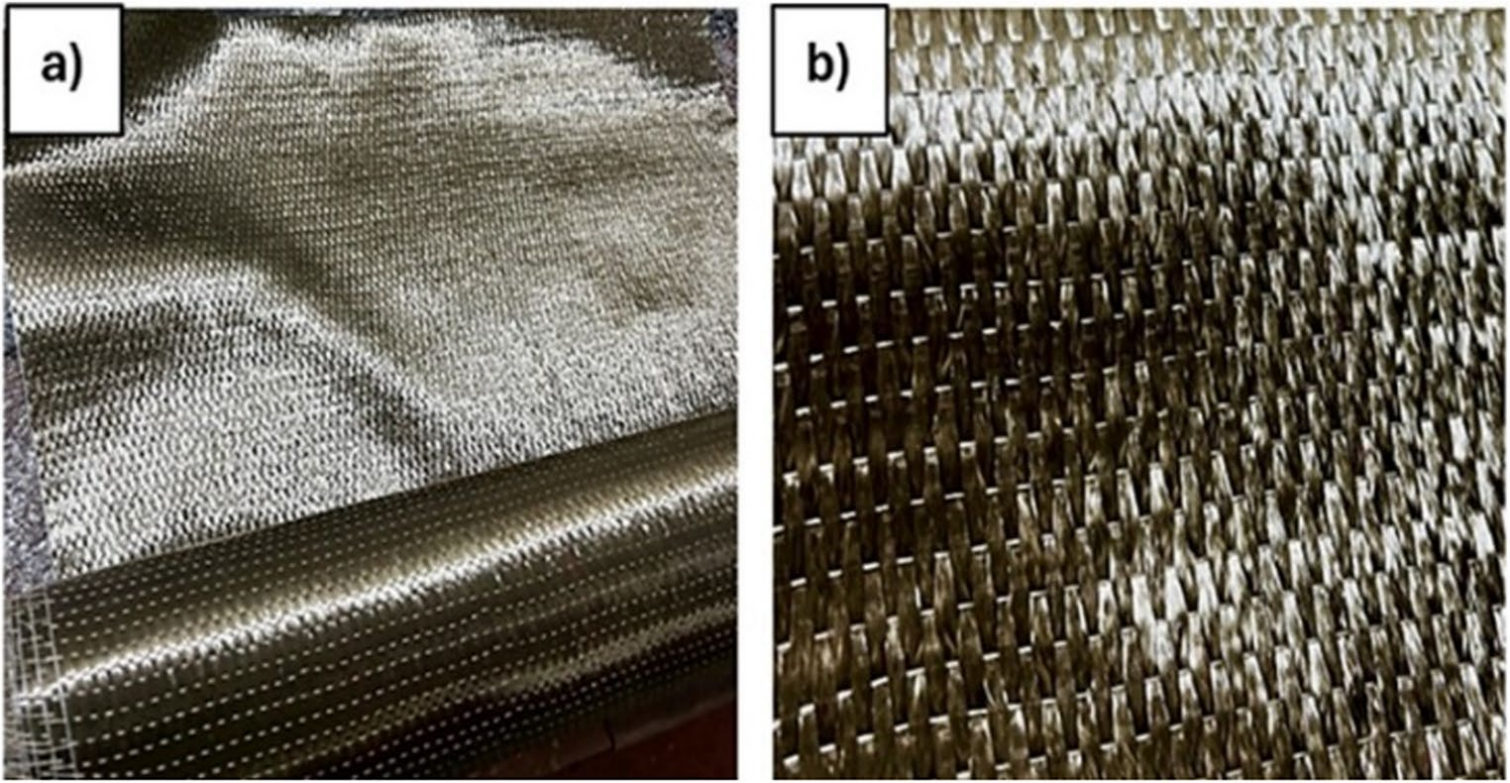
(**a**) Unidirectional basalt fabric (**b**) Closer view of the fabric.

The reinforcement selected for the investigation was a unidirectional basalt fiber obtained from Composites Tomorrow, Gujarat. The material had a GSM of 300, comprising pure basalt fiber yarn arranged in a plain weave pattern. The illustration of the fabric and its closer view are depicted in Fig. 1. The BhorBond^®^ EPCH resin and hardener were used as the matrix material, sourced from Bhor Chemicals and Plastics Pvt. Ltd., India. The chemical composition of the matrix material was Bisphenol-A based liquid thermosetting epoxy resin and low-viscosity amine hardener. The material properties of the matrix phase are presented in Table 2.

**Table 2:** Properties of matrix components.

	Brand	Viscosity	Density
**Epoxy resin**	BhorBond^®^ EPCH	800–1300 CP	1.2 g/cc
**Hardener**	BhorBond^®^ EPCH	450–800 CP	0.95 g/cc

### Composite fabrication

**Figure 2:**
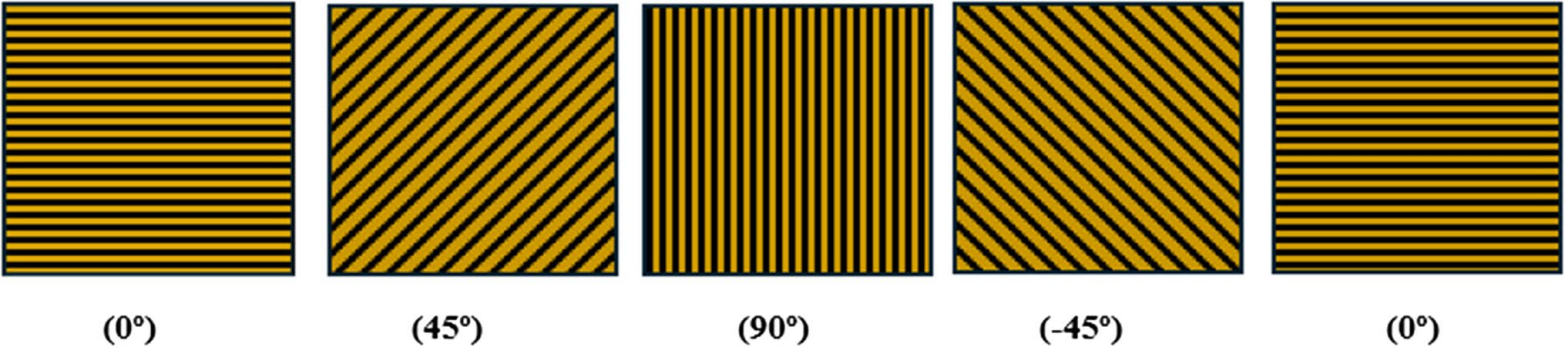
Schematic representation of quasi-isotropic orientation of unidirectional basalt fiber.

The composite laminate, measuring 300 mm×300 mm, was prepared by cutting the fabric into a quasi-isotropic orientation of $\:{[0^\circ\:/45^\circ\:/90^\circ\:/-45^\circ\:/0^\circ\:]}$, which is illustrated in Fig. 2.

**Figure 3:**
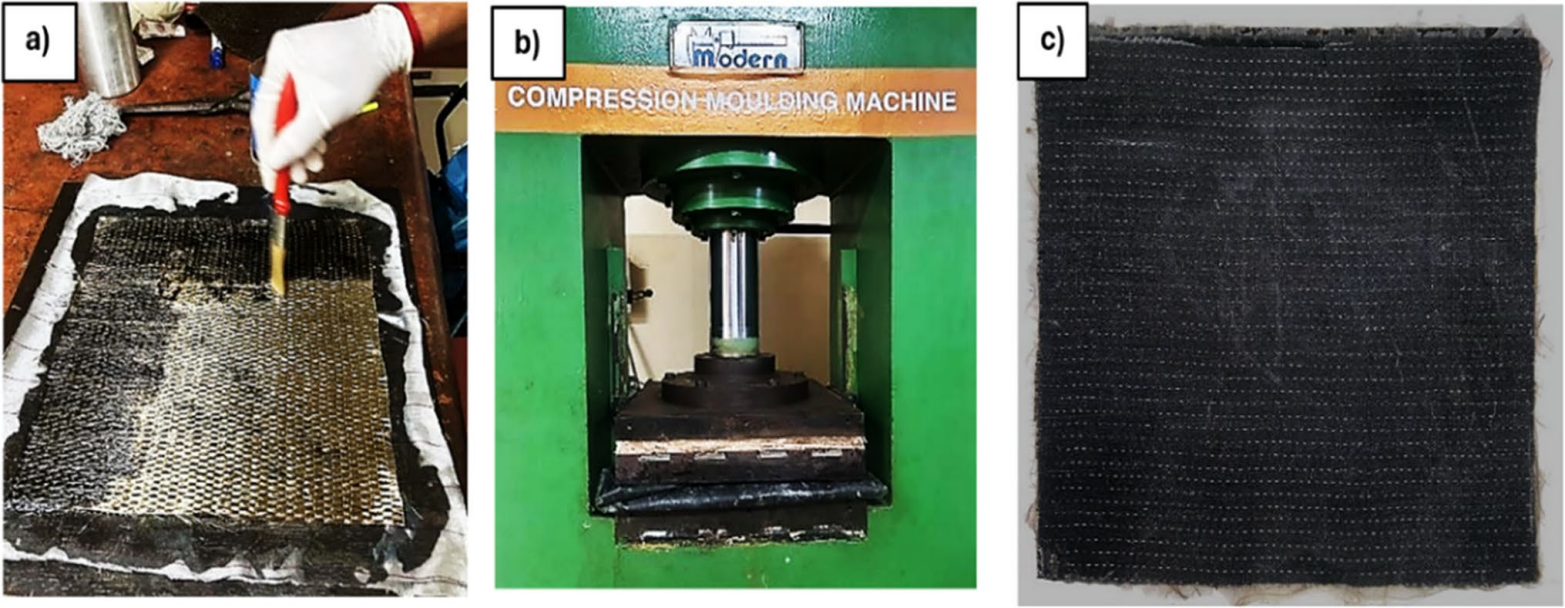
Illustration of (**a**) Hand lay-up method, (**b**) Compression moulding machine, (**c**) laminate post-curing.

The conventional hand layup procedure was employed for fabrication on an open mild steel plate measuring 350 mm×350 mm. The plates were meticulously cleaned and scrubbed with turpentine to eliminate any residual resin and dust, ensuring a pristine, flat surface to prevent bubble formation due to surface irregularities. Both the bottom and top plates were subsequently coated with the release agent prior to fabrication to prevent the cured laminate from adhering to the plates and to facilitate easy removal post curing. The binding matrix was then prepared by mixing the hardener and resin according to the manufacturer’s suggested ratio of 100:38, with a fiber-to-resin ratio of 60:40. The bottom plate was initially coated with the epoxy matrix, followed by the placement of a peel ply. The fabric, cut to the first orientation, was then placed and impregnated or coated with the binding matrix using a brush. The procedure was repeated for all orientations in the specified sequence, with the top plate placed over the laminate. The entire plate assembly was then placed in a compression moulding machine with spacers to attain a uniform thickness of 2.2 mm. The laminate was placed for curing for 24 h at room temperature. Post-curing, Abrasive Water Jet Machine (AWJM), was utilised to cut the laminates in accordance with ASTM and ISO standards. The detailed stages of composite fabrication is presented in Fig. 3.

### Aging procedure

**Figure 4:**
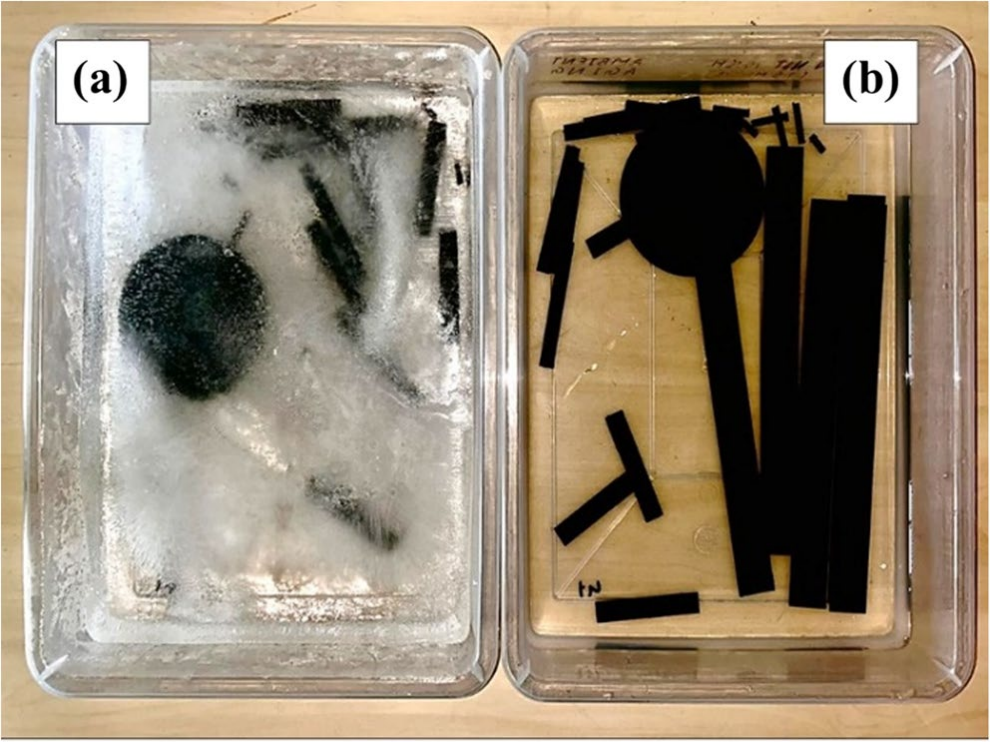
Trays containing (**a**) subzero and (**b**) ambient specimens immersed in distilled water.

A moisture diffusion experiment was conducted till the moisture saturation to assess the moisture absorption characteristics of the laminates. The edges of the specimens were sealed with polymer epoxy to assure that moisture was absorbed evenly throughout the laminate. A high precision digital scale (accuracy of ± 0.001 g) was utilised to measure the initial dry weights of the samples. Five specimens of each test were then categorized into two groups for each aging condition as shown in Fig. 4. Two trays were then filled with distilled water and the following aging conditions were followed:

(i) Ambient aging: Immersion in distilled water maintained at 30 ºC.
(ii) Subzero aging: Immersion in distilled water maintained at -10 ºC in a deep freezer.

To measure moisture gain, the experiment was carried out in accordance with ASTM D5229^45^ standard. Equations 1 and 2 were used to calculate the percentage of moisture absorbed by the specimen at different times and diffusion coefficients respectively.

1 $$M\left(\text{t}\right)\left(\%\right)=\frac{{\text{m}}_\text{t}-{\text{m}}_0}{{\text{m}}_0}\times100$$

Here, M(t) indicates the percentage gain of moisture at time t, $\:{m}_{0}$ is the initial mass of the specimen, and $\:{m}_{t}\:$is the mass at time t.

2 $${D}_{Z}=\pi{\left[\frac{h}{4{M}_{S}}\right]}^{2}\left[\frac{{M}_{2}-{M}_{1}}{\sqrt{{t}_{2}}-\sqrt{{t}_{1}}}\right]=\pi{\left[\frac{h}{4{M}_{S}}\right]}^{2}{k}^{2}$$

Where,$\:{\:\:D}_{Z}$ is the diffusion coefficient, h is the specimen depth, $\:{M}_{1}$ and $\:{M}_{2}$ are the moisture content at times $\:{t}_{1}$and $\:{t}_{2}$, $\:{M}_{S}\:$is the moisture content at saturation, and k represents the slope of the moisture absorption curve.

## Experimental method

### Void fraction

The ASTM D792-20^46^ standard was used to measure the density of the laminate. Samples with dimensions of 10 mm × 10 mm were prepared. The density was determined using Archimedes’ principle. Each sample’s mass was measured with an electronic weighing scale, having a least count of 0.001 g. The volume of the samples was calculated based on the amount of water displaced. The experimental density was then determined by the ratio of mass to volume. Five specimens were selected from different parts of the laminate for testing, and the average density was calculated. The theoretical densities of the laminates were derived using Eq. 3.

3 $${\rho}_{th}=\frac{1}{\frac{{w}_{f}}{{\rho}_{f}}+\frac{{w}_{m}}{{\rho}_{m}}}$$

The void percentage of the laminate was determined using Eq. (4)

4 $$Void\left(\%\right)=\frac{{\rho}_{th}-{\rho}_{ex}}{{\rho}_{ex}}\times100$$

Here, w_f_​ and w_m_​ represent the weight fractions of the fiber and matrix, respectively. ρ_th_​ and ρ_ex_​ correspond to the theoretical and experimental densities, respectively.

### Tensile test set up

**Figure 5:**
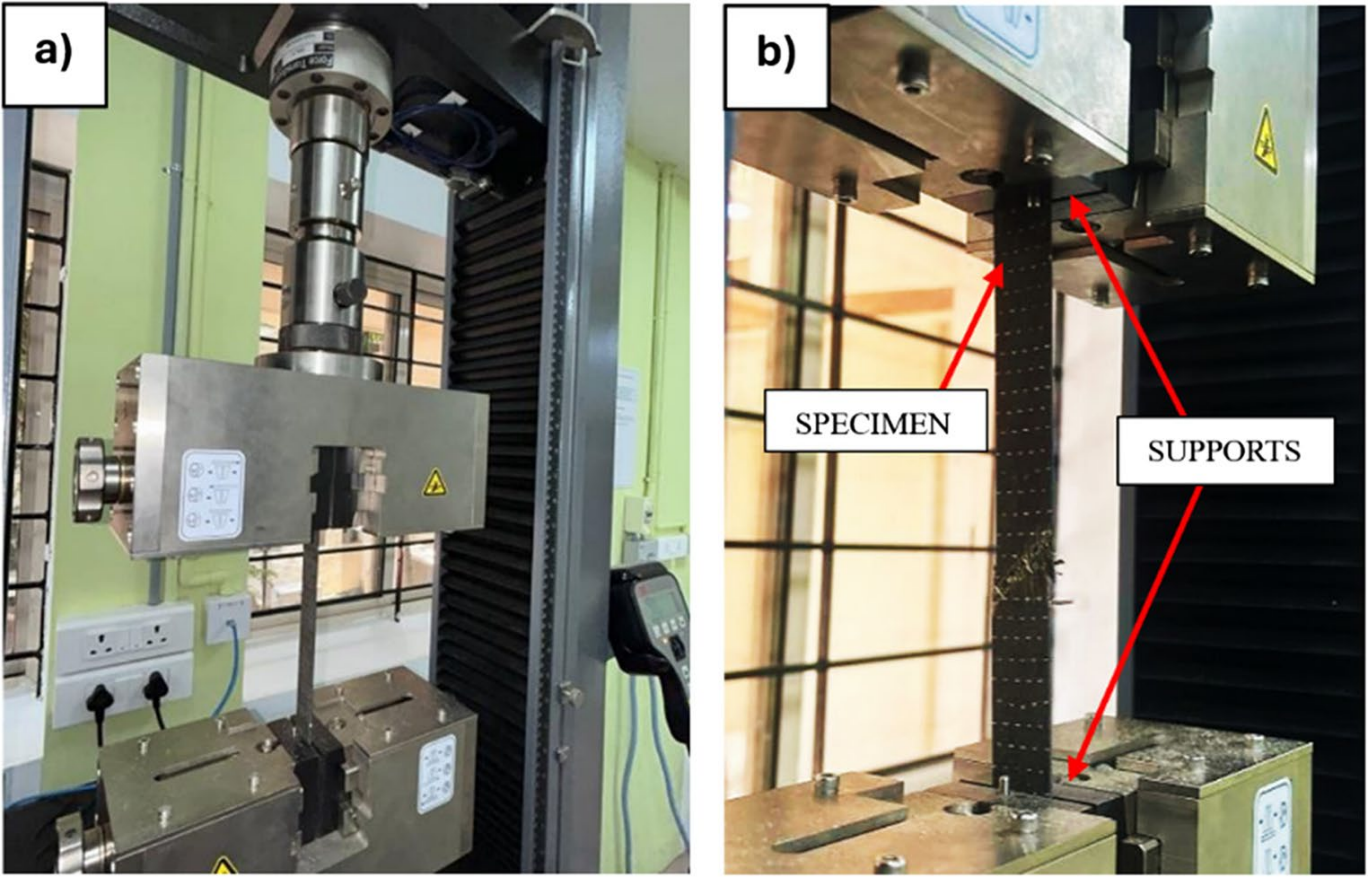
(**a**) Tensile test setup, (**b**) Closer view of the specimen undergoing test.

The tensile test, a type of destructive testing method, is employed to ascertain the strength of a material under tensile load until its breaking or fracture point. This method yields critical information regarding the ultimate tensile strength, maximum tensile load, and tensile strain at failure. Five specimens, each in pristine, ambient, and sub-zero aged conditions, with dimensions of 250 mm×25 mm, were tested in accordance with ASTM D3039^47^ standard. The average of these five values were considered for analysis. The test was executed using the MTS Electromechanical Universal Testing Machine Exceed Model E43, which has a maximum loading capacity of 50 kN as depicted in Fig. 5. The gauge length was set between the grippers at a determined distance of 190 mm, and the specimen was loaded till the failure at a rate of 2 mm/min.

### Flexural test setup

**Figure 6:**
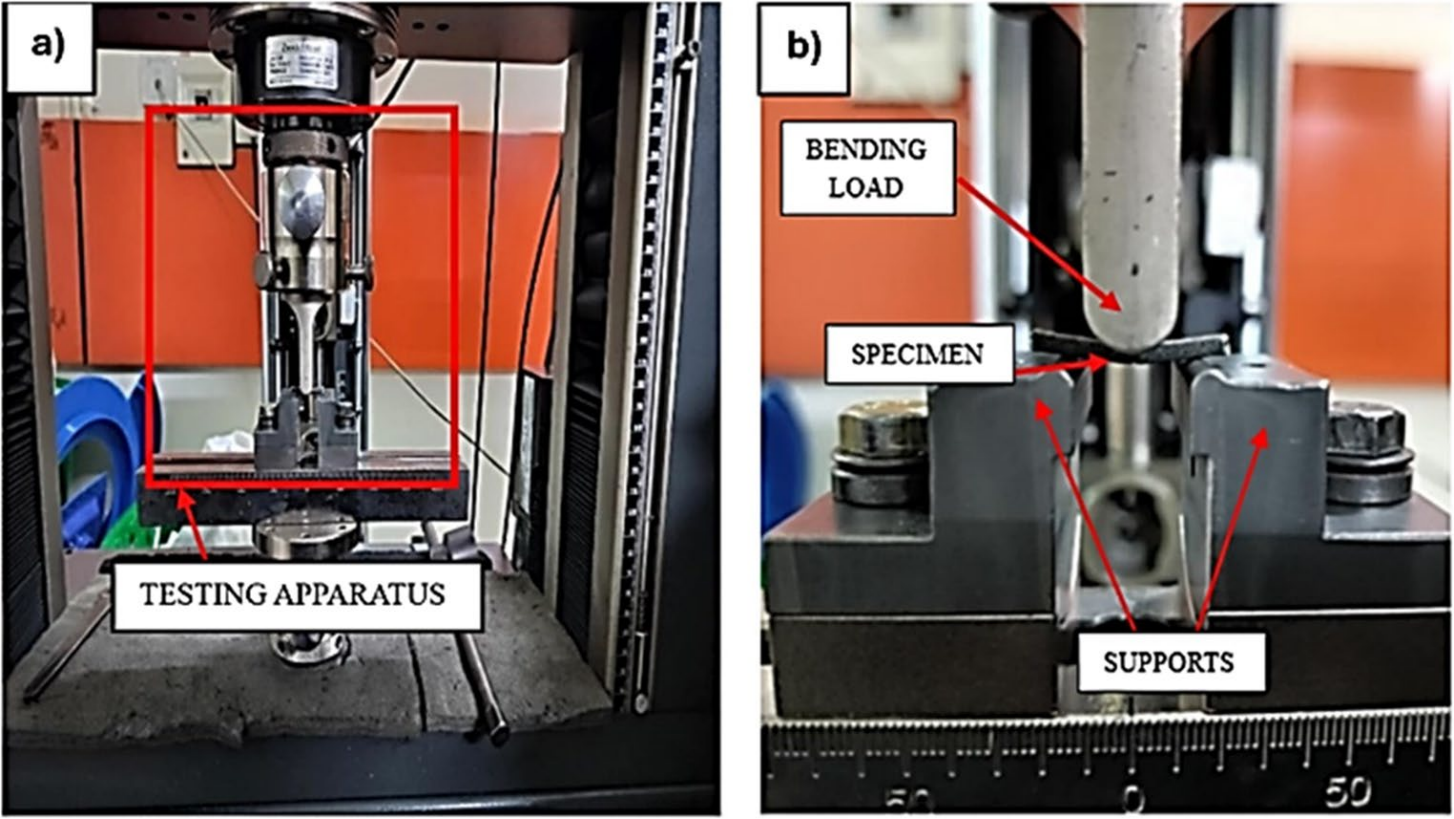
(**a**) Flexural testing apparatus (**b**) Closer view of the specimen undergoing test.

The flexural test is employed to evaluate the extent to which a specimen can bend before undergoing permanent deformation and the test was conducted in accordance with ASTM D7264^48^ standard. The specimen dimensions were 70.4 mm×13 mm, adhering to the standard span to thickness ratio of 32:1 with a standard width of 13 mm. The three-point bending test method was utilised, with supports positioned at the opposite ends of the specimen and the loading applied at the midspan. The test was performed using the MTS Electromechanical Universal Testing Machine Exceed Model E43, which has maximum loading capacity of 50kN as depicted in Fig. 6. The gauge length between the supports was maintained at 32 mm, and a head displacement rate of 2 mm/min was applied until failure. The loading and displacement after testing are determined and subsequently converted to flexural stress$\:\:\left({\sigma\:}_{f}\right)$ and flexural strain $\:\left({\upepsilon\:}\right)$ using Eqs. 5 and 6.

5 $${\sigma}_{f}=\frac{3FL}{2b{d}^{2}}$$

6 $${\upepsilon}=\frac{6\text{D}\text{d}}{{\text{L}}^{2}}$$

Where F is the load applied at mid-span, L is the support span, b is the width of the specimen, d is the thickness, and D denotes the maximum central deformation of the specimen.

### Short Beam Shear (SBS) test

**Figure 7:**
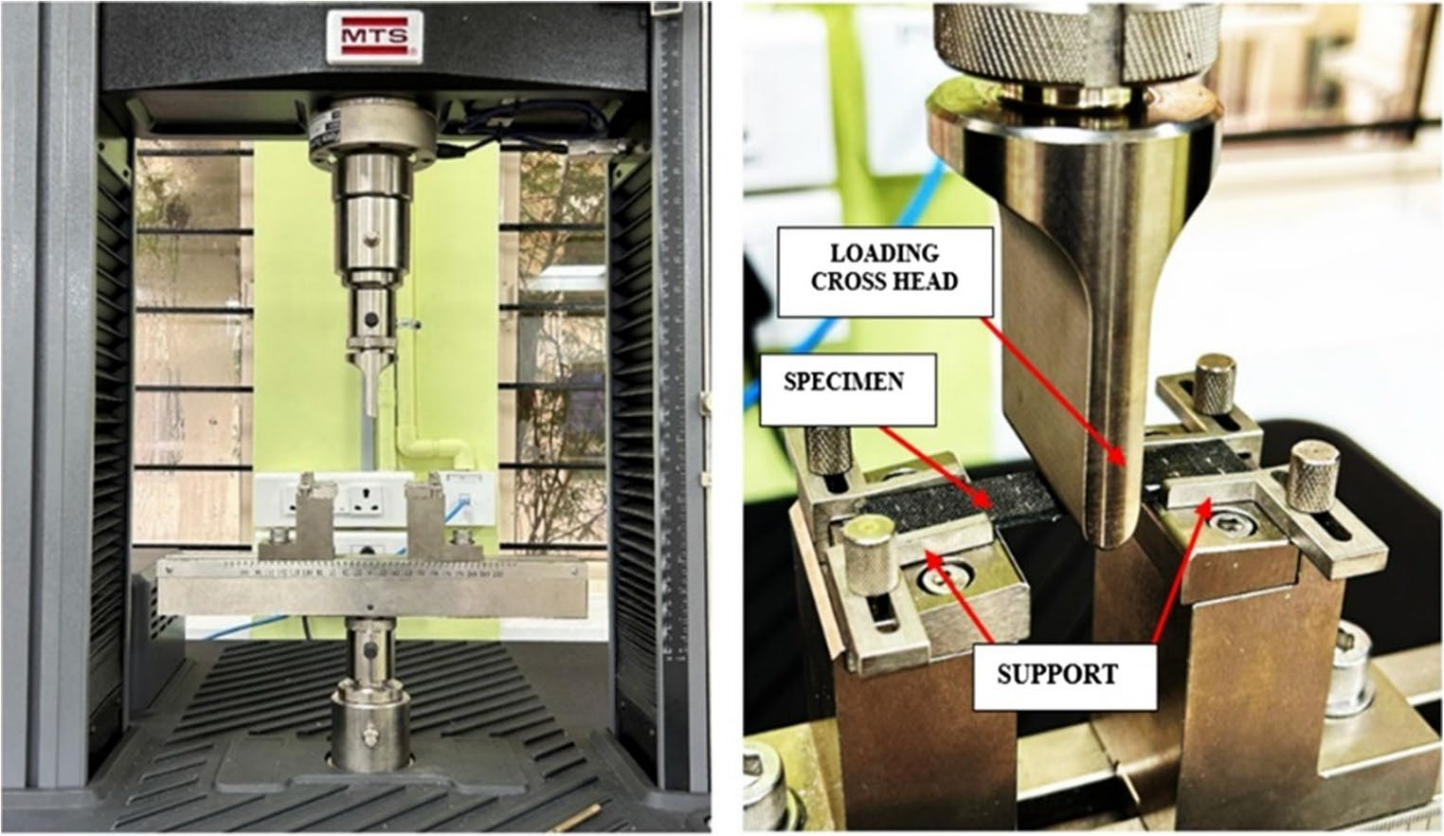
SBS test set up.

The SBS test is crucial for assessing shear strength in laminate composites, helping to predict and prevent delamination failures. The test was conducted using the Zwick/Roell HIT 50P which has a maximum loading capacity of 20 kN to evaluate the bonding strength between the layers of the laminate. The specimens were prepared according to ASTM D2344^49^ standards, with dimensions of 25 mm × 4.4 mm. The test was carried out with a span length of 16 mm and a head displacement rate of 2 mm/min as shown in Fig. 7. The machine functions by applying shear forces between the layers and measures the shear strength, providing critical data on the materials interlaminar adhesion properties.

### Charpy impact test

**Figure 8:**
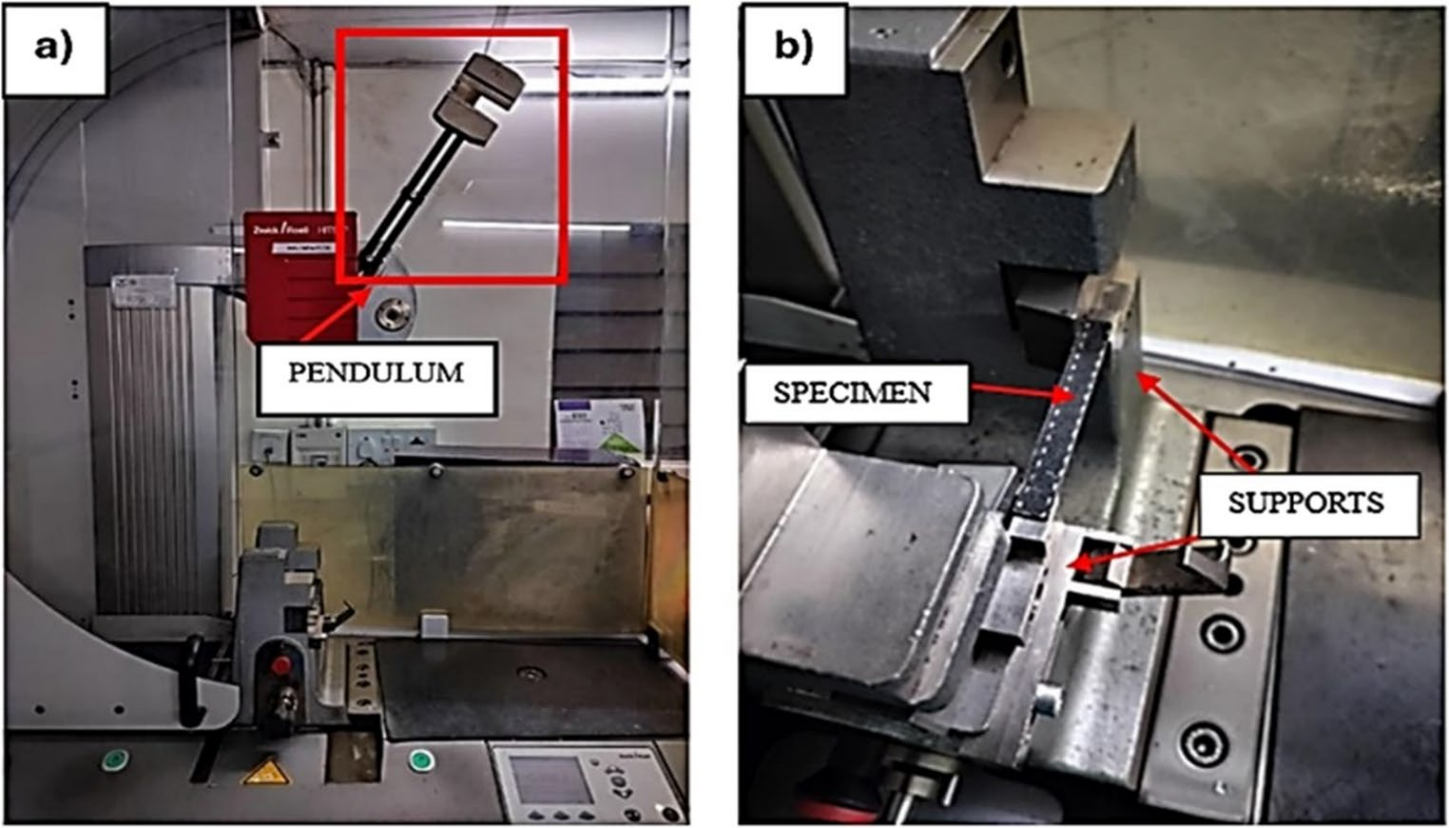
(**a**) Impact test setup, (**b**) Closer view of the specimen.

The Charpy impact test is essential for measuring a material’s toughness and resistance to impact-induced fractures. The five specimens of pristine and each aging condition, with dimensions 80 mm×10 mm was tested in accordance with ISO 179 − 11^50^ standard. The average values from these tests were considered for analysis. Testing was performed using a Zwick/Roell Hit 50P machine, which has a theoretical impact velocity of 3.807 m/s and working capacity of 7.5 J. The testing apparatus comprises a pendulum with high stiffness and concentrated mass, which is raised to a specified height and then allowed to strike a designated point on the specimen, as illustrated in Fig. 8. The specimens were secured such that the point of impact was centered along a span length of 32 mm.

### Vibration test

**Figure 9:**
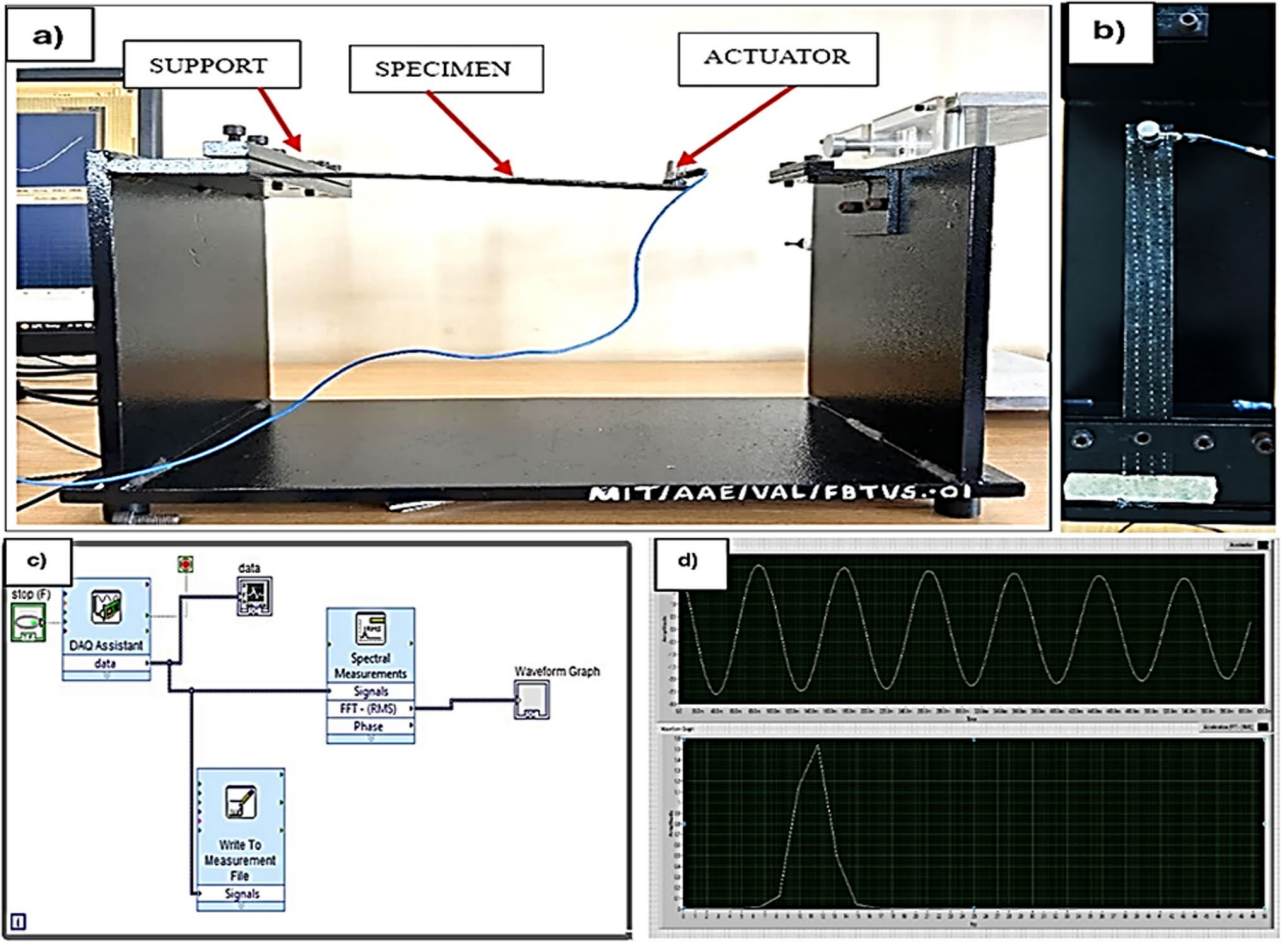
(**a**) Impact Hammer test setup (**b**) Closer view of specimen (Top view) (**c**) LabView DAQ simulation (**d**) acceleration vs. frequency and acceleration vs. time plot as seen in LabView.

Vibrational analysis acts as a crucial tool for comprehending the structural integrity of materials under diverse loading conditions and environments, providing insights into damping characteristics such as damping ratio, stiffness, and natural frequencies. The damping characteristics were assessed in accordance with ASTM E756-05^51^ standard. Specimens for this test were prepared to the dimensions of 250 mm×25 mm. As depicted in Fig. 9, the specimen was fixed at one end in the cantilever beam setup, while the other end was left free. A PCB Piezotronics accelerometer, with a sensitivity of 106.3 mV/g, was connected to the free end of the specimen. Random free vibrations were then induced in the specimens, and the stiffness (K), storage modulus ($\:{E}_{s}$), and damping ($\:{\upzeta\:}$) were determined using the following Eqs. 7, 8, and 9

7 $$k=\frac{3{E}_{s}I}{{L}^{3}}$$

8 $${f}_{n}=\frac{1}{2{\uppi}}\sqrt{\frac{\text{k}}{\text{m}}}$$

9 $${E}_{s}=\frac{16{{\uppi}}^{2}{\text{f}}_{\text{n}}^{2}\text{m}{\text{L}}^{3}}{\text{b}{\text{h}}^{3}}$$

These equations provide crucial data for understanding the material’s response to dynamic loading conditions and its potential for structural applications. The logarithmic decrement and damping ratio are determined by utilising the logarithmic decrement method. Logarithmic decrement ($\:\delta\:$) is a measure of the rate at which oscillations decrease in amplitude overtime in a damped system and it is calculated by measuring the amplitudes of two consecutive peaks in the damped oscillatory system, in which $\:{X}_{1}$, is the first peak, and $\:{X}_{2}$ is the second peak, respectively by using Eqs. 10,

10 $$\delta=\frac{1}{n}{ln}\left(\frac{{X}_{1}}{{X}_{2}}\right)$$

The damping ratio (${\upzeta}$) is a dimensionless nature which describes how oscillations in a system diminish following a disruption and is connected to the logarithmic decrement by Eqs. 11,

11 $${\upzeta}=\frac{{\updelta}}{\sqrt{4{{\uppi}}^{2}+{{\updelta}}^{2}}}$$

### Impedance tube test

Transmission loss is defined as the reduction in acoustic energy when sound propagates through a material. It is quantified as the difference in sound pressure levels between the incident and transmitted sound, expressed in decibels (dB). This parameter is essential in acoustics for evaluating and designing materials that effectively block or reduce sound transmission.

**Figure 10:**
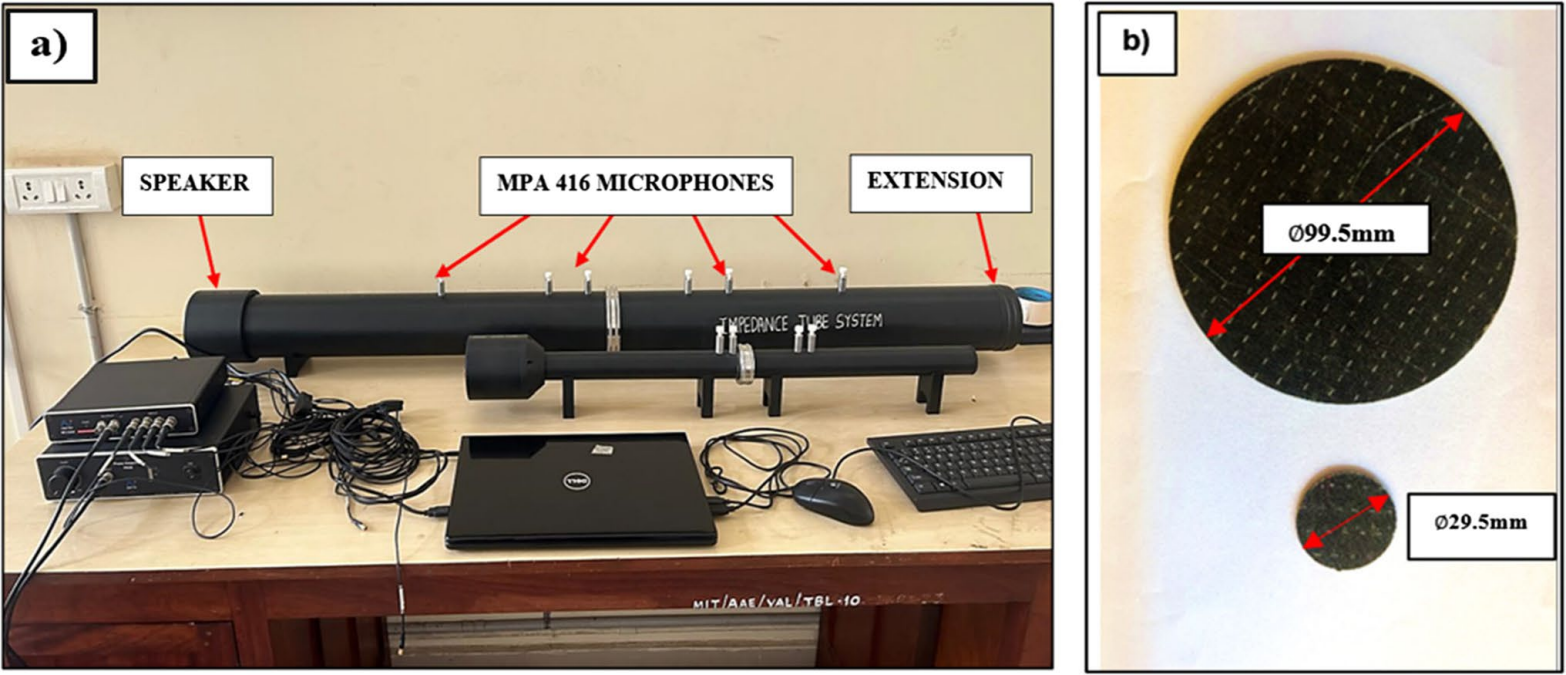
(**a**) Impedance tube apparatus (**b**) Closer view of specimens.

To measure transmission loss, the impedance tube test, as outlined by BSWA technology, was employed. ISO 10534-2^52^ standards were used to evaluate circular specimens with diameters of 99.5 mm and 29.5 mm as shown in Fig. 10. During the test, the loudspeaker produces noise, and the specimens serve as sound barriers, absorbing some portion of the sound. Four microphones with varying sensitivities were strategically positioned in both the large and small tubes during different intervals. These intervals were maintained for a duration of ten minutes, utilising the transfer function method across a frequency range from 63 Hz to a high frequency of 6300 Hz. The data obtained from the large tube with wide spacing and the small tube were amalgamated to construct a graph illustrating transmission loss versus frequency, as assessed through the impedance tube test. This process enabled the determination of the transmission loss values. This method provides a comprehensive understanding of the materials effectiveness in attenuating sound across different frequencies. The data obtained from this method is crucial for developing materials with greater acoustic properties, which will allow for better sound insulation and noise reduction in a variety of applications.

## Results and discussion

### Void content

Voids are unfilled spaces within composite materials, where polymers and fibers fail to completely occupy the structure. These voids act as a key indicator of the quality of fabrication, as higher void content typically suggests imperfections in the manufacturing process. The presence of voids can significantly affect the mechanical properties of the composite, such as its strength, stiffness, and fatigue resistance, potentially leading to premature failure in critical applications. The theoretical density of the laminate was determined to be 1363 kg/m³, while the experimental density was found to be 1337 kg/m³. As a result, the calculated void percentage was 1.94%, which is within acceptable limits.

### Moisture absorption behaviour

**Figure 11:**
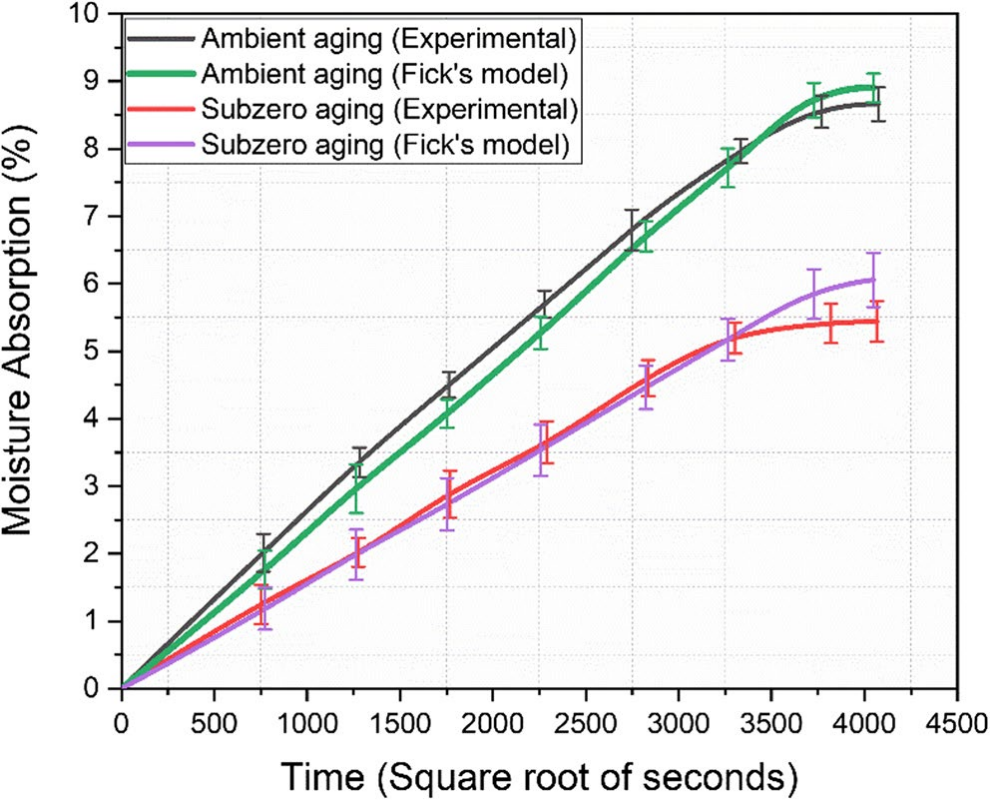
Moisture absorption behaviour of aged specimens.

The experimental values for moisture absorption rates of basalt composites under ambient and subzero aging conditions are depicted in Fig. 11. The graph illustrates the degree of moisture absorption by the samples over the immersion time.

The graph clearly shows that composites aged under ambient and subzero conditions exhibited distinctly different behaviors, both demonstrating Fickian characteristics. The samples soaked in distilled water at ambient temperatures, assumed to have 100% relative humidity, exhibited the highest moisture content at 8.66%, while the laminates aged under subzero conditions absorbed 5.44% moisture. The ambient-aged samples exhibited a gradual weight increase throughout the aging process, with an estimated weight gain of 0.7% per week, reaching moisture saturation after 162 days. In contrast, the subzero-aged specimens achieved moisture saturation after 139 days. Table 3 presents the outcomes of moisture diffusion test for the two aging conditions.

**Table 3:** Results of moisture absorption test.

Aging condition	Moisture at saturation (%)	Coefficient of diffusion $D_z\left(mm^2/sec\right)$
**Ambient**	8.66%	1.45×10^-7^
**Subzero**	5.44%	1.01×10^-7^

The variation in water absorption between the samples can be explained by Fick’s law, which states that diffusion occurs in response to a concentration gradient, represented by the change in concentration across a specific area. The diffusion coefficient, D_z_​, expressed in mm^2^/sec, serves as the proportionality constant. The concentration gradient is a key factor driving the moisture absorption process in polymer composites^53^. Initially, a high gradient causes rapid diffusion of water molecules into the composite. As the material nears saturation, the gradient gradually decreases, eventually leading to equilibrium. The ambient condition generates a maximum concentration gradient between the external environment and the composite. This leads to the rapid diffusion of water molecules into the composite to equalize the concentration difference, resulting in a steeper slope and the highest moisture content and absorption rate at saturation. Moisture absorption was comparatively slower under subzero conditions due to lower temperatures, which reduced molecular mobility and caused the formation of physical barriers by ice. Additionally, the polymer matrix stiffened, leading to a lower diffusion coefficient. These factors collectively hindered the movement of water molecules, resulting in slower moisture uptake^54^.

Moisture absorption in fiber-reinforced polymer composites is influenced by several factors, including the hygroscopic nature of polymers, void content, the presence of microcracks, fiber-matrix interactions, and various diffusion mechanisms^55^. Both basalt and epoxy are hydrophilic, naturally attracting and absorbing moisture from their environment. However, the extent of absorption was moderately reduced due to the quasi-isotropic orientation, which limits moisture ingress pathways, and the high cross-link density of the epoxy resin, which decreases the free volume within the resin. This makes it more challenging for water molecules to penetrate and diffuse through the material. A highly cross-linked polymer network provides enhanced resistance to moisture absorption^56^.

### Tensile behaviour

**Figure 12:**
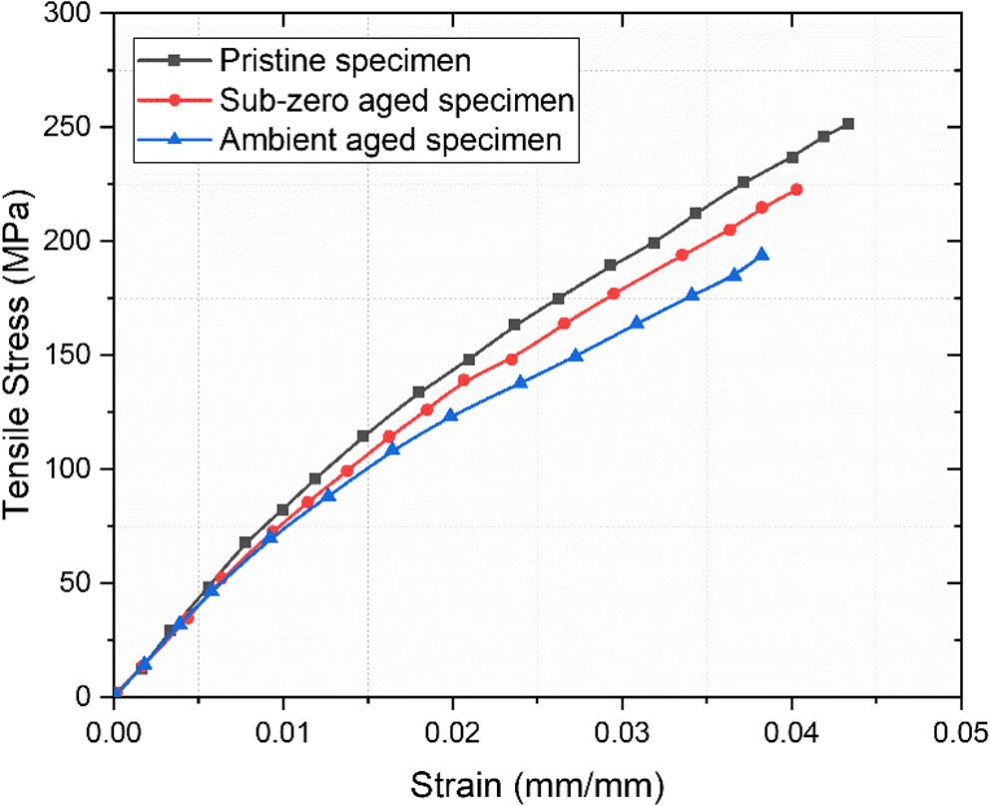
Tensile stress-strain curve for pristine and aged specimens.

The tensile properties of pristine, ambient aged, and subzero aged basalt fiber reinforced polymer composites were thoroughly evaluated. The results, including ultimate tensile strength, maximum loading, strain rate, and Young’s modulus, are systematically tabulated in Table 4. The tensile properties followed the order of pristine > subzero > ambient. The tensile stress-strain curves for these three conditions are illustrated in Fig. 12.

**Table 4:** Tensile results for pristine, ambient, and subzero aged specimens.

Specimen type	Max. tensile Load (kN)	Ultimate tensile strength (MPa)	Tensile strain at failure (mm/mm)	Young’s modulus (GPa)
**Pristine**	13.01	236.70	0.043	5.635
**Subzero**	11.27	205.02	0.038	5.35
**Ambient**	9.87	179.48	0.035	4.82

**Figure 13:**
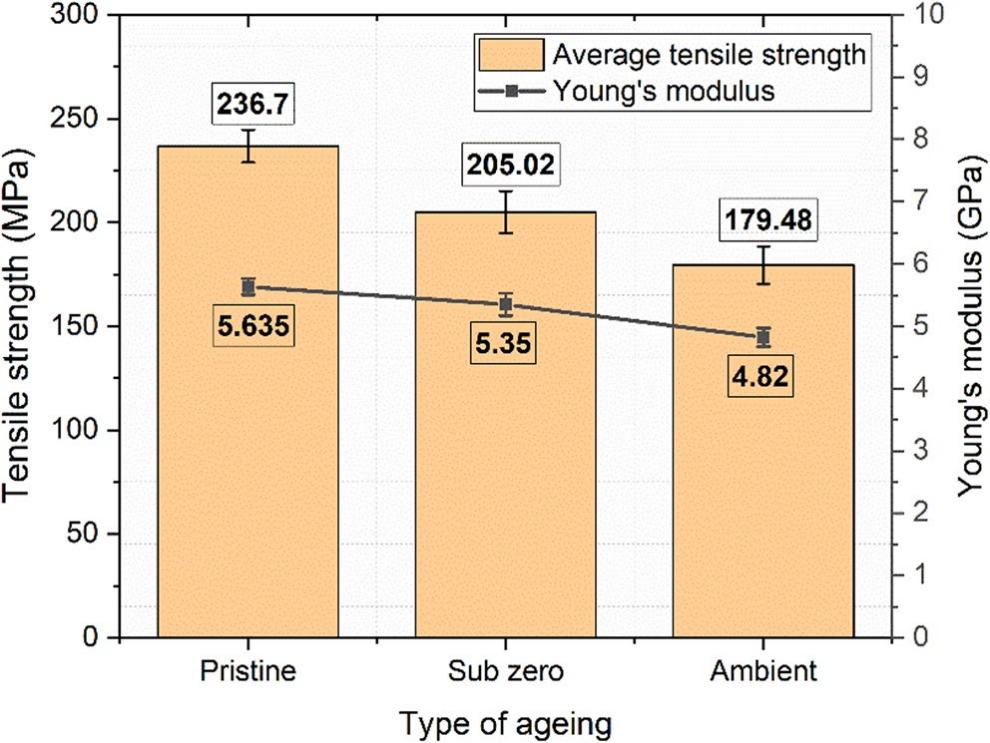
Ultimate tensile stress and modulus of unaged and aged samples.

The maximum tensile strength and Young’s modulus were found to be 236.7 MPa and 5.63 GPa, respectively for pristine specimens. The subzero-aged specimens showed a moderate tensile strength of 205.02 MPa and a modulus of 5.35 GPa, while the ambient-aged specimens demonstrated the lowest tensile strength of 179.48 MPa and a modulus of 4.82 GPa, as shown in Fig. 13.

The specimens exposed to ambient conditions experienced a significant reduction in tensile strength, decreasing by 24.17% compared to the pristine specimens. In contrast, those subjected to subzero conditions showed a relatively smaller reduction of 13.38%. The degradation observed in the ambient-aged specimens is primarily due to moisture absorption, which causes swelling of the polymer matrix. Inorganic polymeric materials are especially susceptible to high moisture absorption, leading to swelling and degradation of their mechanical properties. During the immersion period, matrix generally absorb more water than the fibers, weakening the interfacial bonding and reducing the material’s ability to withstand tensile loads, making it more prone to failure. Water acts as a plasticizer and can hydrolyze the interface, breaking the chemical bonds between the fibers and the matrix. This results in reduced efficiency of load transfer from the matrix to the fibers, thereby decreasing the overall mechanical properties^57^.

**Figure 14:**
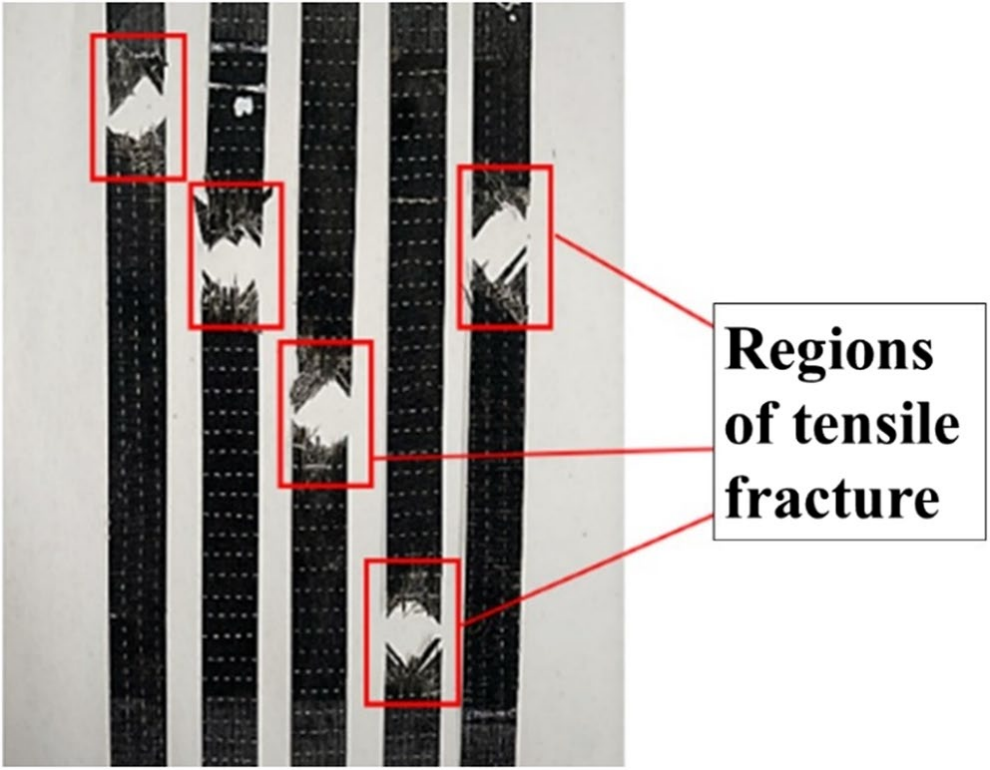
Fractured tensile specimens.

Furthermore, the chemical composition of the resin significantly influences its interaction with water molecules. Resins containing polar groups or those capable of forming hydrogen bonds with water are more prone to moisture absorption. In subzero conditions, ice formation can enhance interfacial bonding between the fiber and matrix, while the quasi-isotropic fiber distribution helps mitigate the decline in tensile properties. Additionally, fabrication defects such as surface irregularities, epoxy-rich regions, and microbubbles can promote moisture retention, thereby reducing the composite’s structural integrity and durability^58,59^. Therefore, the reduced tensile characteristics of basalt composites are significantly impacted by aging conditions. Pristine samples exhibited the highest resistance to tensile stress and deformation, while ambient-aged specimens show the greatest degradation. The fractured tensile specimens are as shown in Fig. 14.

### SEM analysis

**Figure 15:**
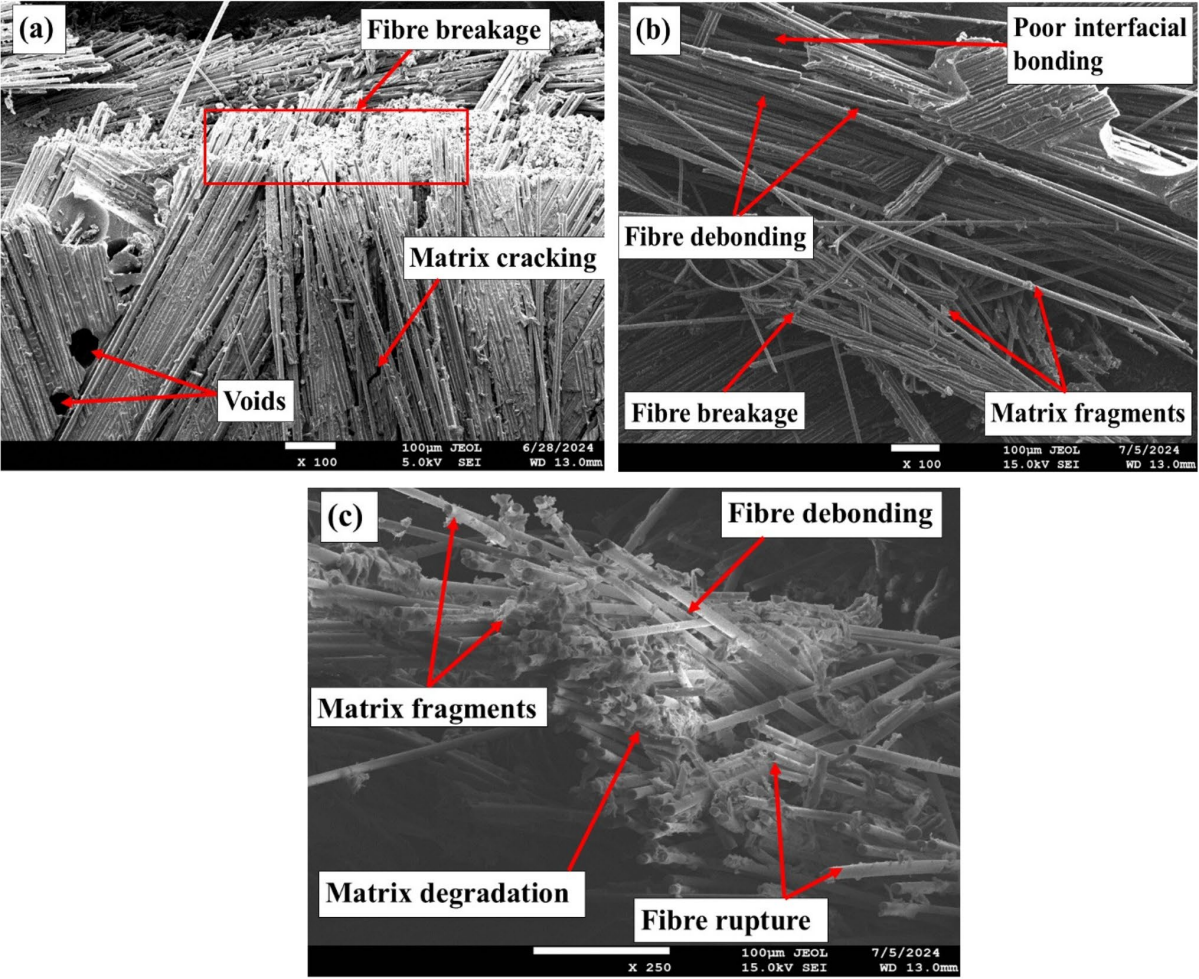
SEM images of fractured tensile samples: (**a**) in the pristine condition, (**b**) after ambient aging, and (**c**) following sub-zero aging.

Figure 15 presents SEM images of fractured pristine and aged tensile specimens. SEM analysis of the pristine specimens revealed several distinct fracture phenomena. Notably, fiber breakage was observed, where fractures displayed brittle surfaces with sudden, clean breaks, indicating failure of the fiber with minimal elastic deformation. This behavior suggests that the fibers could withstand high tensile loads up to the breaking point. Another significant observation was matrix cracking, appearing as relatively smooth, clean breaks on the fracture surface, indicative of brittle failure. Since the matrix is designed to bind and transfer loads between the fibers, its brittle nature suggests that it is more likely to fail suddenly and with little plastic deformation. The smooth cracks and minimal breakage highlight the matrix’s lack of toughness, making it susceptible to sudden failure under tensile stress.

SEM images of tensile fracture specimens subjected to ambient aging revealed several critical failure mechanisms, including fiber debonding, poor interfacial bonding, fiber rupture, matrix cracking, micro matrix cracking, and the presence of matrix fragments. Fiber debonding indicates inadequate load transfer between the fibers and the matrix, which increases the susceptibility to tensile failure. Prolonged environmental exposure leads to matrix swelling, contributing to void formation and exacerbating poor interfacial bonding, ultimately resulting in weakened adhesion and facilitating premature failure. The occurrence of fiber rupture further diminishes the composite’s reinforcing capacity, as the load is redistributed to the remaining intact fibers and matrix, which may be insufficient to bear the applied stress. Additionally, both matrix cracking and micro matrix cracking, along with matrix degradation, significantly compromise the composite’s overall performance due to the detrimental effects of ambient aging. In contrast, subzero specimens primarily exhibit fiber rupture with relatively better fiber-matrix interaction, reduced debonding, and fewer matrix fragments compared to ambient-aged specimens, although they do not perform as well as pristine samples.

### Flexural behaviour

Table 5 presents the average values for ultimate flexural strength, failure strain, and modulus for pristine, ambient-aged, and subzero-aged conditions. During the test, the loading nose applies force at the mid-span of the specimen, creating compression on the top surface and tension in the bottom layers.

**Table 5:** Flexural test results of pristine and aged specimens.

Specimen type	Ultimate strength (MPa)	Failure strain (mm/mm)	Modulus (GPa)
**Pristine**	385.192	0.030	11.92
**Subzero**	291.83	0.069	10.71
**Ambient**	327.17	0.095	9.83

**Figure 16:**
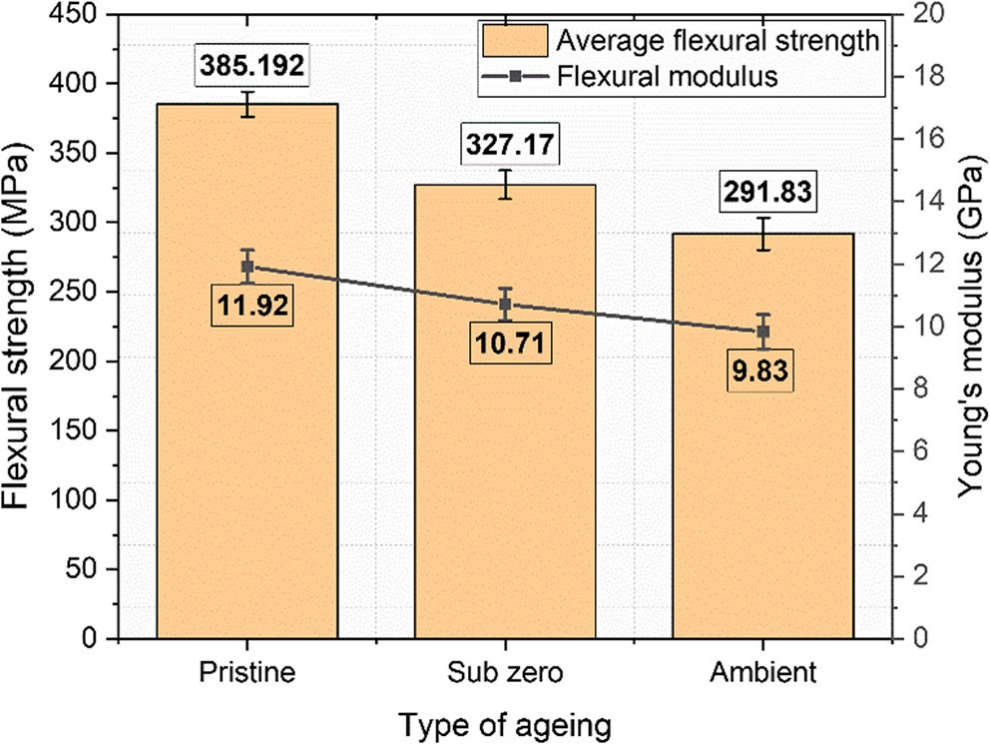
Flexural stress and modulus of unaged and aged samples.

The ultimate flexural strength was highest in the pristine specimens at 385.19 MPa, followed by the subzero-aged specimens at 327.17 MPa, and the ambient-aged specimens at 291.83 MPa as shown in Fig. 16.

**Figure 17:**
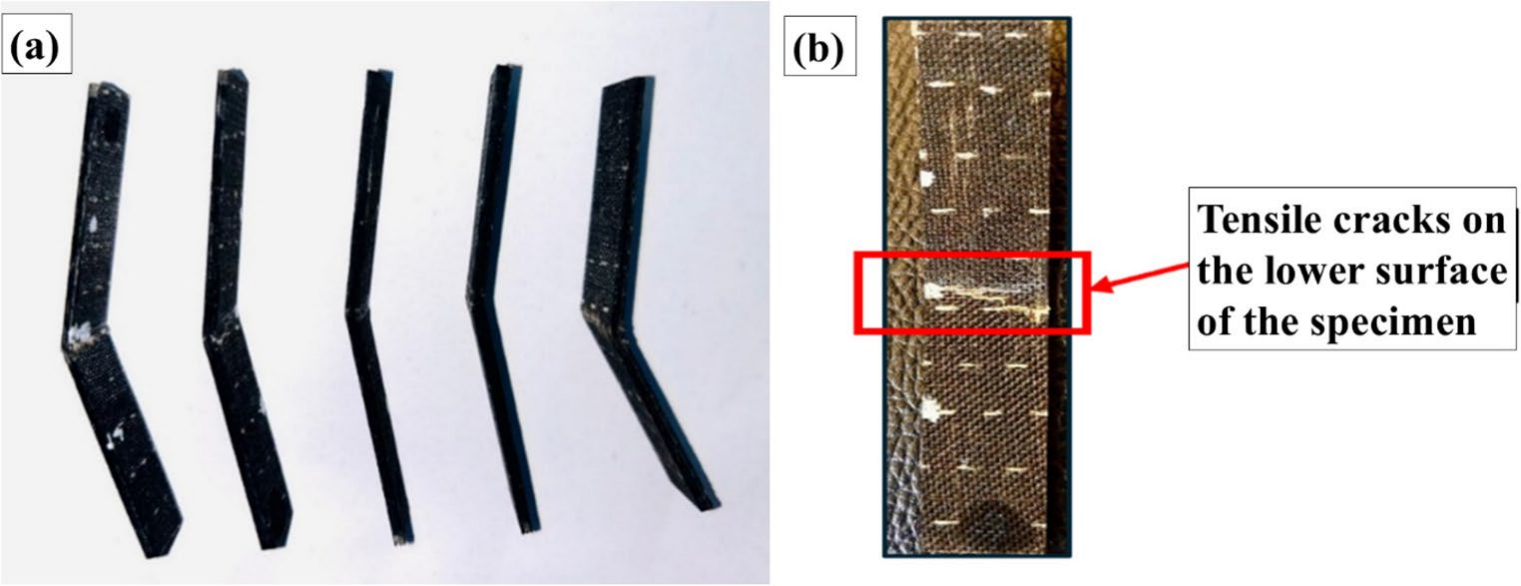
(**a**) Fractured flexural specimens, (**b**) Close view of fracture region.

The flexural strength of both subzero and ambient-aged specimens was lower compared to the pristine specimens, with flexural strength retention rates of 68.3% and 37.6%, respectively. The flexural modulus followed the order: pristine > subzero > ambient, indicating a greater decline in the ambient-aged specimens compared to the subzero-aged specimens. The fractured specimens did not break into two pieces; instead, bent into a V shape as shown in Fig. 17. Cracks were observed on the compression side of the fractures, while the tension side displayed stretched and broken fibers at the mid-span of the specimen.

Moisture exposure was identified as the primary cause of reduced flexural performance, leading to swelling of the matrix and basalt fibers. As immersion time increases, small fissures and voids within the composite become more prominent, creating localized areas with steep concentration gradients. Water molecules diffuse into these areas, weakening the bond between the fiber and matrix, resulting in delamination and a reduced gradient^60,61^.

Moreover, an interesting observation was that the ductility of the material increased due to moisture content, making the epoxy resin more elastic. As a result, the pristine and subzero samples were relatively more brittle compared to the ambient-aged samples. The subzero samples exhibited better properties than the ambient samples even after aging, due to ice acting as a plasticizer. At subzero temperatures, the polymer matrix becomes stiffer and more brittle, reducing the material’s ability to accommodate moisture penetration. Capillary action, which facilitates moisture ingress through microchannels and voids, is less effective when water is in its solid form compared to its liquid form. The cohesive forces in ice are much stronger than those in liquid water, thus helping to retain the mechanical properties.

### SEM analysis

**Figure 18:**
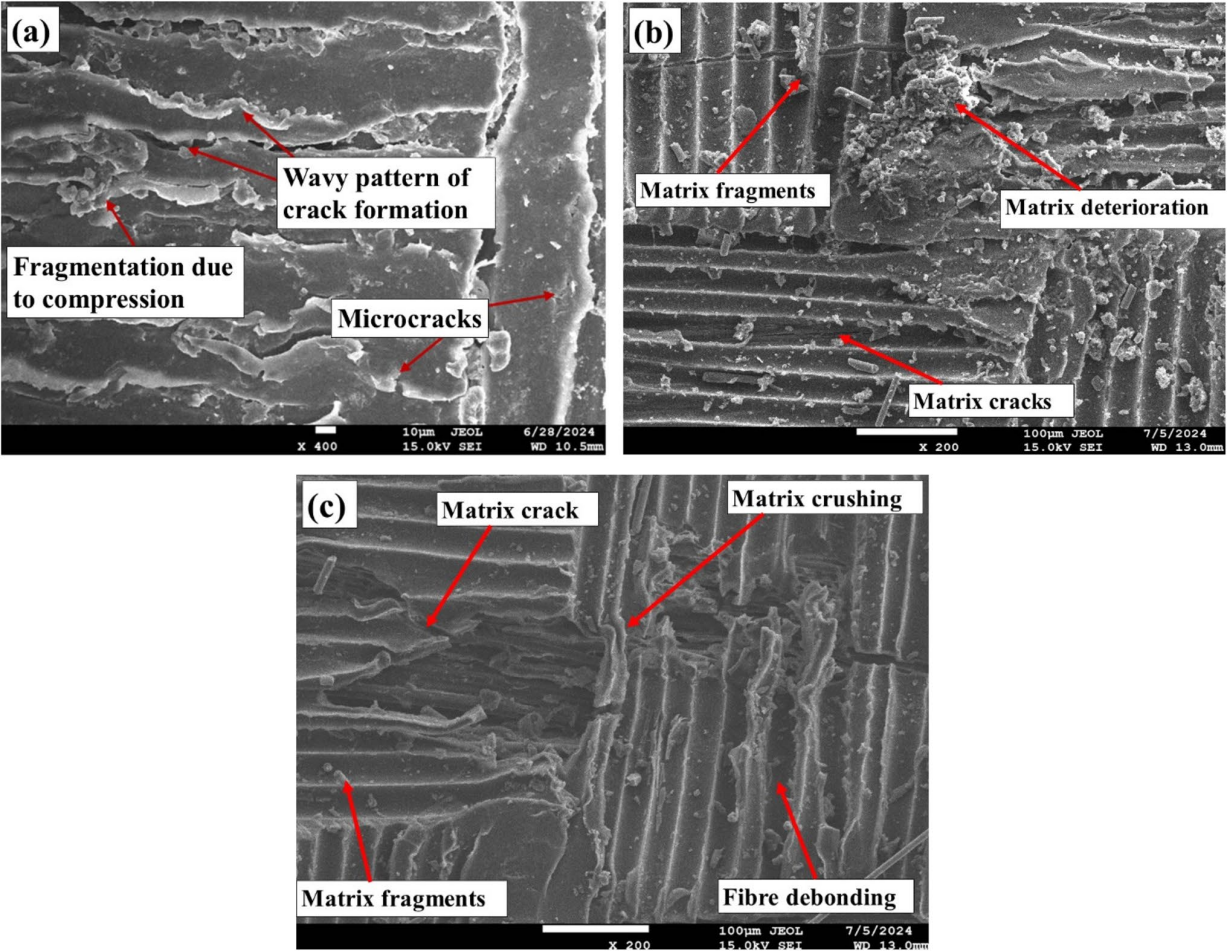
SEM images of fractured flexural samples: (**a**) in the pristine condition, (**b**) after ambient aging, and (**c**) following sub-zero aging.

Figure 18 displays SEM images of fractured pristine and aged tensile specimens. The SEM analysis of flexural fractures in pristine composites revealed the occurrence of microcracks throughout the matrix, indicating the onset of failure under stress. A distinctive wavy pattern of matrix cracks is observed, suggesting a non-uniform distribution of stress during flexural loading. This pattern indicates the material’s attempt to redistribute the applied load to alleviate localized stress concentrations. There were minimal visible signs of matrix fragmentation, demonstrating the matrix’s resistance to compression. However, compression buckling is evident, showing that the matrix deforms before failing. This buckling results from compressive forces acting on the matrix, creating localized zones of instability where the matrix fails to maintain structural integrity. This phenomenon highlights the complex interaction between the matrix and fibers, where the matrix primarily absorbs compressive loads, and any defects or weaknesses can lead to localized failure and subsequent crack propagation.

SEM observations of the ambient-aged flexural specimens revealed marked deterioration, characterized by extensive matrix fragmentation, crushing, and the presence of matrix micro cracks. This indicates severe damage under flexural loading, with the matrix showing signs of deterioration and disintegration, which compromises its ability to withstand applied stresses. The visible fiber breakage suggests that the fibers, too, failed to support the loads, undermining the composite’s structural integrity. Additionally, protruding matrix fragments due to compressive forces point to a loss of cohesiveness under load. The occurrence of cracks within the matrix further emphasizes the compromised state of the composite, likely resulting from the combined effects of environmental degradation and mechanical stress. Subzero-aged flexural fractured specimens exhibit distinct features compared to ambient-aged specimens. These specimens demonstrate reduced matrix fragmentation, indicating better preservation of the matrix material under flexural loading at low temperatures. Additionally, there is less fiber debonding and matrix crushing observed, which suggests improved interfacial bonding and structural integrity. Although some fiber rupture is present, the occurrence of micro matrix cracks is minimal, further reflecting the enhanced performance of the composite in subzero conditions. Overall, the combination of reduced damage mechanisms highlights the superior resilience of subzero-aged specimens compared to their ambient-aged counterparts.

### Short beam shear strength

**Figure 19:**
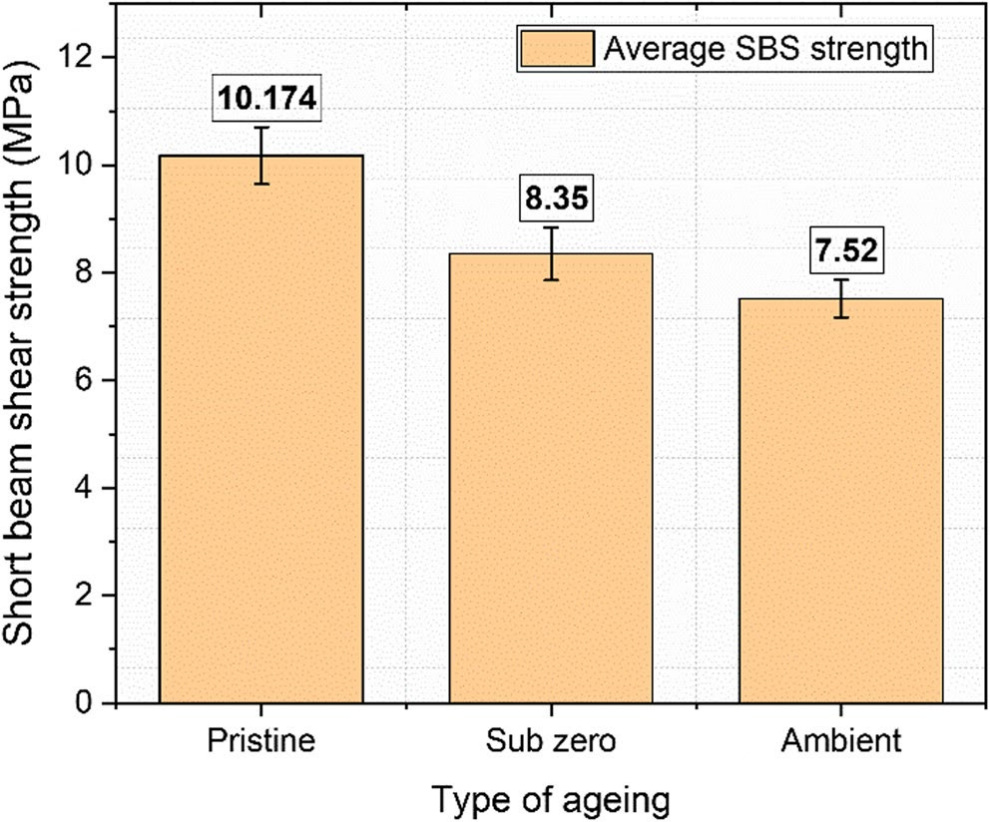
SBS strength results for pristine and aged samples.

The Short Beam Shear (SBS) test is utilized to assess the interlaminar shear strength of composite materials. In this test, the applied load is carefully designed to minimize tensile and compressive forces, thereby concentrating on inducing shear stresses between the layers of the laminate. Among the samples tested, the pristine specimen exhibited the highest interlaminar shear strength at 10.17 MPa. This was followed by the subzero and ambient samples, which demonstrated shear strengths of 8.35 MPa and 7.52 MPa, respectively as shown in Fig. 19. Compared to the pristine samples, which are assumed to retain 100% of their original strength, the subzero samples showed a strength reduction of 17.92%, while the ambient samples exhibited the greatest strength reduction at 26.08%. Consequently, the strength retention for the subzero and ambient specimens was 82.08% and 73.92%, respectively.

SBS specimens, due to their smaller size, are particularly vulnerable to environmental exposure and aging. This increased susceptibility results in more pronounced changes in mechanical properties compared to other types of specimens. Similar to tensile and flexural specimens, fibers in SBS specimens exposed to aging facilitate the diffusion of water molecules and their interaction with the matrix. This interaction weakens the interfacial forces and bonding between the fiber and the matrix. Over time, water molecules disrupt interchain Van der Waals forces and hydrogen bonds, leading to the plasticization of the resin. Prolonged immersion time leads to several phenomena, including micro-buckling, the development of hygroscopic residual stresses from differential thermal expansion, and osmosis. These effects disrupt the transmission of shear forces between the fibrous layers, weakening interfacial cohesion and lowering the glass transition temperature. In subzero conditions, the freezing process releases latent heat, causing localized temperature variations within the composite. This results in uneven moisture distribution and absorption rates, further impacting the material’s performance^62,63^.

### Impact behaviour

**Figure 20:**
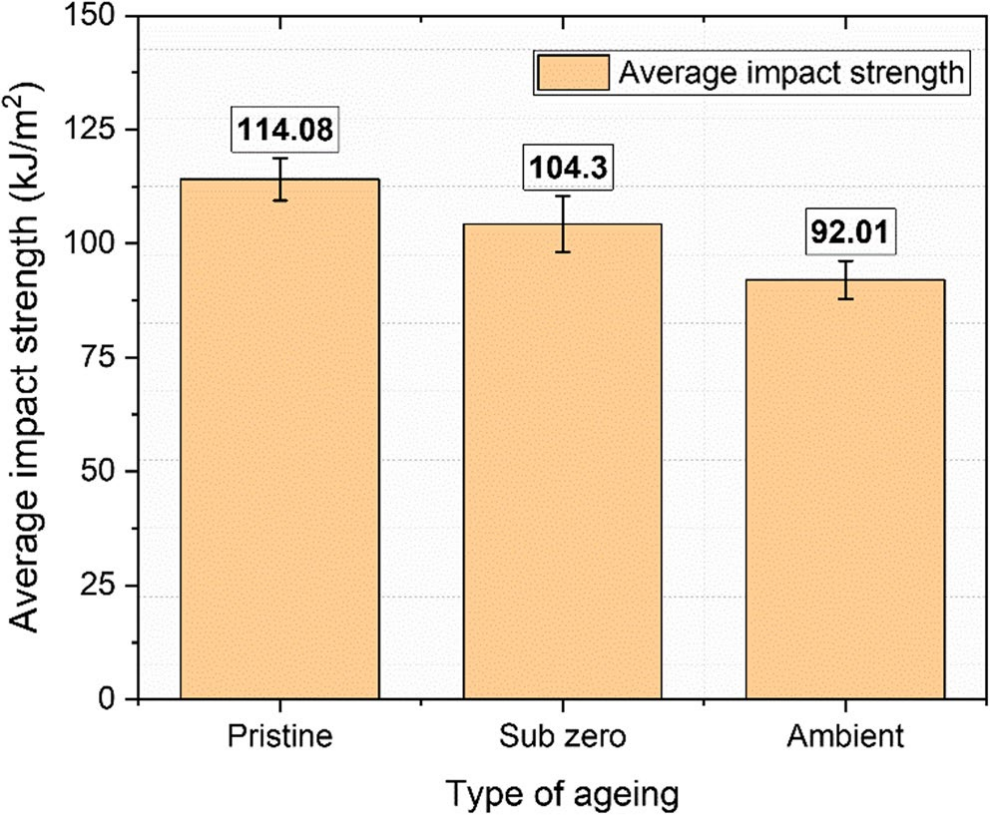
Impact strength results for pristine and aged samples.

The Charpy impact test is an economical and effective method for measuring the impact energy absorbed during the phase of irreversible deformation in fiber-reinforced polymer composites. Due to their brittle nature, these composites typically exhibit minimal elastic deformation, with the thermosetting polymer matrix primarily absorbing the impact force. As illustrated in Fig. 20, the pristine samples demonstrated the maximum impact strength at 114.08 kJ/m^2^, followed by the subzero and ambient specimens, with values of 104.3 kJ/m^2^ and 92.01 kJ/m^2^, respectively.

The reductions in impact strength for the subzero and ambient aged samples were determined to be 8.57% and 19.34%, primarily due to moisture exposure from water. The hygroscopic nature of the basalt fibers and the epoxy-hardener mix led to fiber-matrix separation. The impact strength retention for the aged samples, compared to the pristine sample, was found to be 91.43% for the subzero samples and 80.6% for the ambient samples. Ambient-temperature immersion resulted in a notable reduction in impact strength, attributed to extensive water absorption that caused matrix swelling, softening, and degradation.

Role of ice formation at subzero temperatures in the degradation of impact properties of basalt fiber composites is significant. When moisture within the composite freezes, it expands, generating internal stresses that can initiate microcracking, particularly at the fiber-matrix interfaces and within the matrix itself. Over time, these microcracks can propagate, leading to embrittlement, which reduces the material’s ductility and toughness. Consequently, the combined effects of microcracking and embrittlement can substantially degrade the composite’s mechanical properties, especially its impact resistance, making it more prone to failure under stress.

### SEM analysis

**Figure 21:**
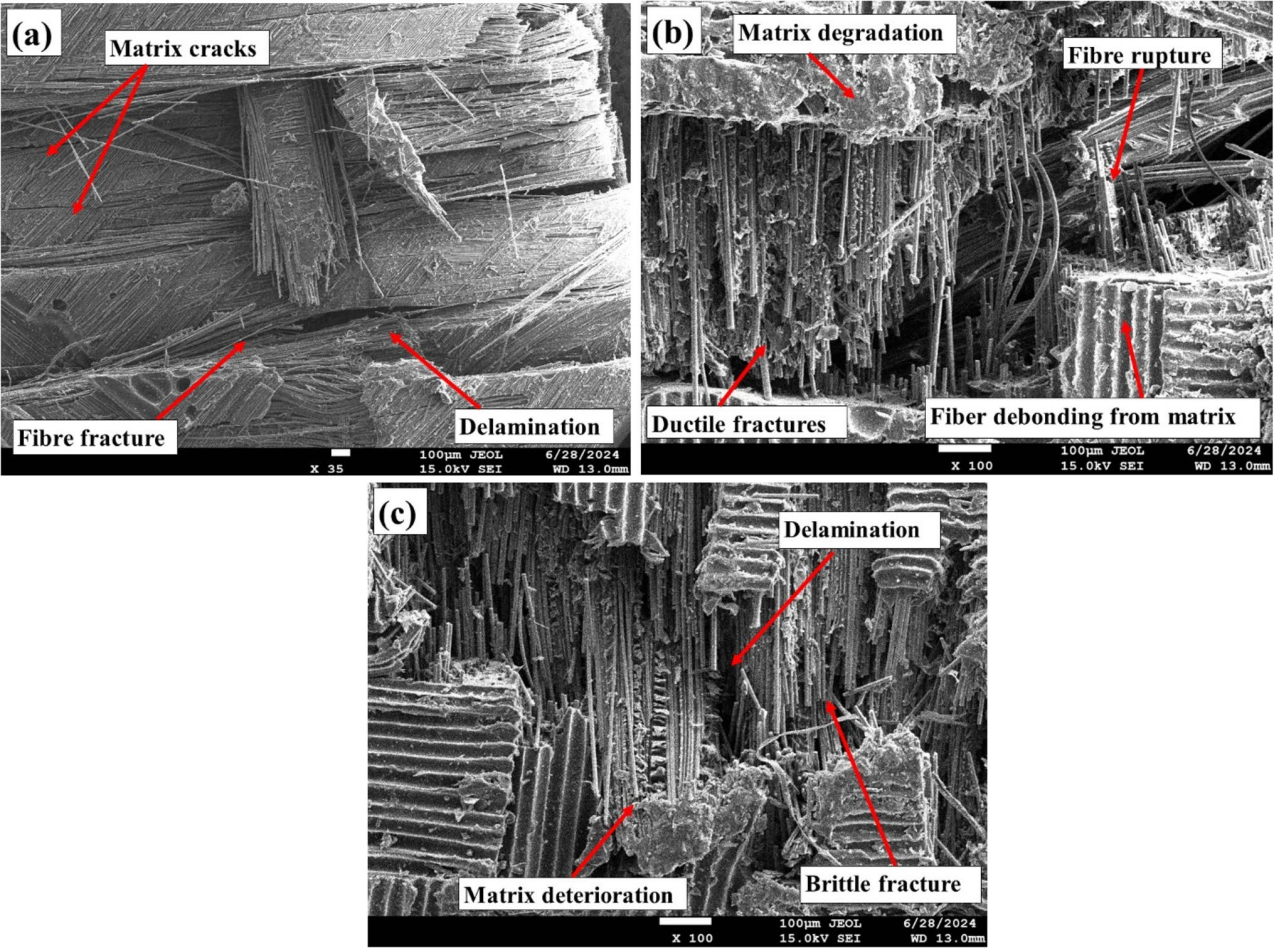
SEM images of fractured impact samples: (**a**) in the pristine condition, (**b**) after ambient aging, and (**c**) following sub-zero aging.

Figure 21 shows the SEM images of fractured impact specimens. Fracture analysis indicated that ambient-aged specimens exhibited prominent fiber-matrix debonding, fiber rupture and delamination, all of which are indicative of compromised structural integrity due to moisture-induced weakening. The analysis also revealed signs of matrix degradation and fiber fractures, further contributing to the overall deterioration of the material. While some ductile fracture characteristics were present, the predominant failure mechanisms highlight the vulnerability of the composite under these conditions, emphasizing the detrimental effects of moisture on its performance.

Conversely, composites immersed at sub-zero temperatures showed minimal reduction in impact strength compared to pristine specimens. The reduced water absorption at lower temperatures, combined with the formation of ice within the matrix, resulted in less severe degradation. Fracture patterns in sub-zero specimens predominantly displayed brittle fractures with less matrix-related damage and fewer instances of delamination. This suggests that while freezing introduces stress that can cause brittle fractures, it does not lead to the same extent of matrix deterioration observed at ambient temperatures. These findings highlight the significant impact of temperature on moisture absorption and the resultant fracture behavior of composite materials.

### Vibrational analysis

**Figure 22:**
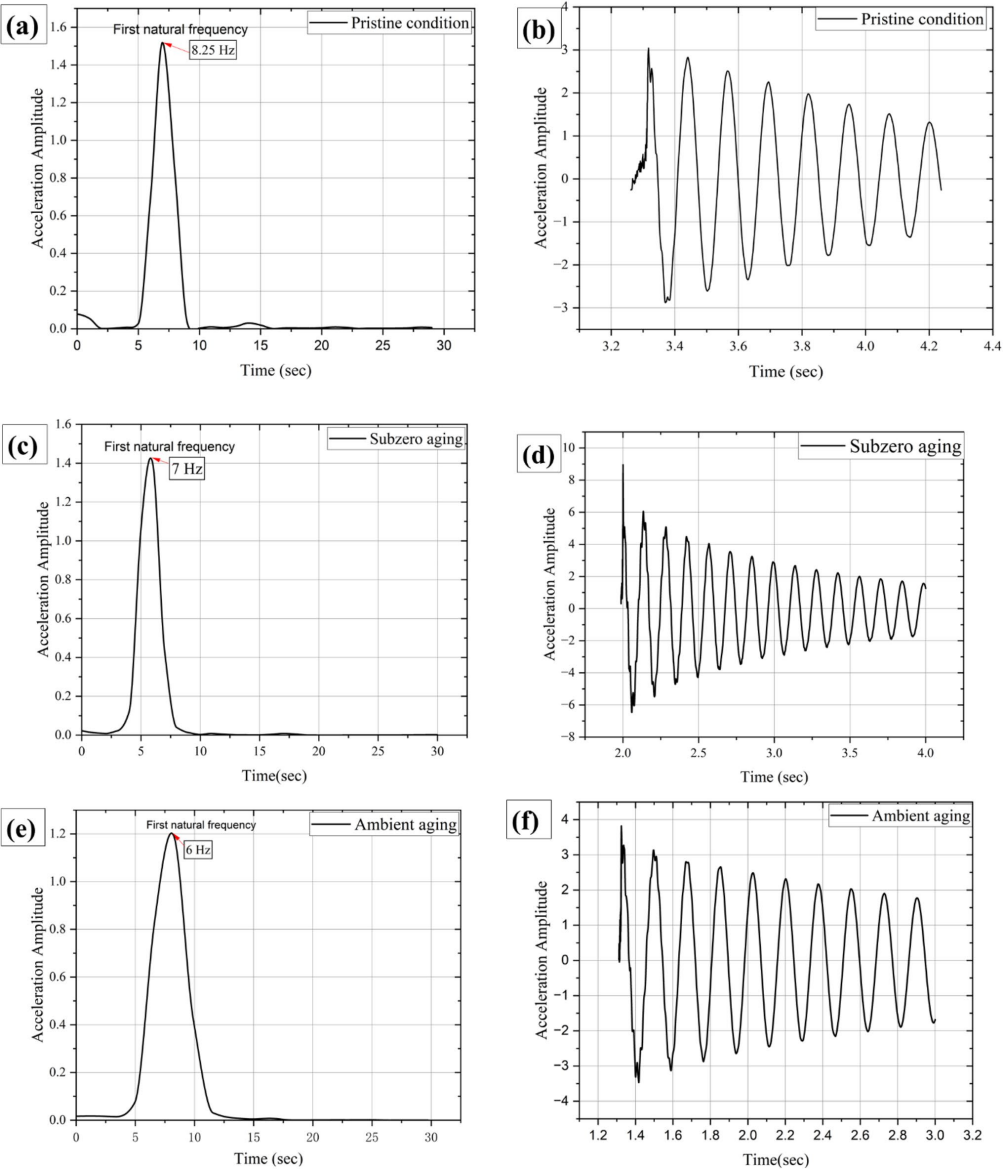
Natural frequencies and damping analysis graphs for (**a**, **b**) pristine, (**c**, **d**) subzero, and (**e**, **f**) ambient aged specimens.

The impact hammer test is used to analyze the dynamic behavior of a structure by providing information on its frequency response and damping characteristics. The procedure involves striking the specimen with a hammer to generate energy impulses while simultaneously measuring the resulting vibrations with an accelerometer. Using a data acquisition device, the relationship between the impulse signal and the object’s response can be analyzed through a graph of the natural frequencies of the structure. Figure 22 illustrates the damping behavior and natural frequency plots for all conditions. The vibrational properties, including natural frequency, stiffness coefficient, storage modulus, logarithmic decrement, and damping ratio for all samples, are summarized in Table 6.

**Table 6:** Vibrational analysis test results.

Aging Condition	Natural frequency, ${f}_{n}$ (Hz)	Stiffness Coefficient K (*N*/m)	Storage Modulus ($\:{E}_{S}$) (GPa)	Logarithmic Decay (δ)	Damping Ratio (ξ)
Pristine	8.25	32.56	6.02	0.23	0.036
Subzero	7.0	23.20	4.29	0.38	0.060
Ambient	6.0	17.68	3.27	0.215	0.034

From the table, it is observed that the pristine samples demonstrated the highest set of vibrational property values for natural frequency, stiffness coefficient and storage modulus followed by subzero and ambient condition respectively. The pristine sample exhibited the maximum natural frequency of 8.25 Hz followed by subzero and ambient having 7 Hz and 6 Hz, respectively. Compared to the pristine sample, subzero recorded the lowest percentage reduction in natural frequency by 15.15% whereas the ambient specimen recorded the highest reduction in natural frequency by 27.27%. The deterioration in natural frequencies in these quasi-isotropic basalt specimens can be determined by Eq. 12 of the Meirovich’s continuous beam model^64^.

12 $${\text{f}}_{\text{n}}\text{=}\frac{1}{{2\pi}}\frac{{\text{(1.875)}}^{\text{2}}}{{\text{L}}^{\text{2}}}\sqrt{\frac{\text{EI}}{\mathrm\rho\text{A}}}$$

Where L represents the overall length, $\:{E}_{S}\:$being the modulus of the material, $\:\rho\:\:$ being the density, I being the second moment of area of the specimen and A denotes the area of cross section. The equation demonstrates that the material stiffness is directly proportional to the natural frequency. According to the relation between the damping ratio and the stiffness of the material as seen in Eqs. 13,

13 $$\zeta=\frac c{2\sqrt{km}}$$

The damping ratio is inversely to the stiffness of the material. Higher the value of k, lesser the damping ratio, better is the material in absorbing the vibrations.

Further, from the table, it is witnessed that the subzero samples exhibited the highest value of damping ratio of 0.060 followed by the pristine and ambient specimens, having 0.036 and 0.034. The subzero specimen demonstrated the highest logarithmic decay compared to the pristine and ambient aged specimens.

Moisture absorption generally leads to a decrease in the characteristic natural frequencies of fiber-reinforced polymer structures. The natural frequency is also affected by the structure’s stiffness and mass. When moisture is absorbed by the composite, the mass increases slightly, resulting in a reduction in natural frequency. Vibrational tests on aged composites typically show a decrease in natural frequencies compared to their dry counterparts. This reduction is more pronounced in materials with significant moisture uptake, as observed in the ambient specimens.

The primary factors contributing to reduced stiffness and, consequently, lower natural frequencies are matrix plasticization and weakened fiber-matrix interfaces. These effects are more significant than the mass increase due to moisture absorption. Moisture can cause hydrolytic deterioration of the polymer matrix, breaking down chemical bonds and further reducing stiffness. Additionally, moisture absorption lowers the glass transition temperature (T_g_) of the polymer matrix, making it more flexible and further reducing overall stiffness. However, for subzero specimens, the brittleness of the epoxy and the higher cross-link density of the polymer have resulted in improved damping properties.

### Sound absorption behaviour

**Figure 23:**
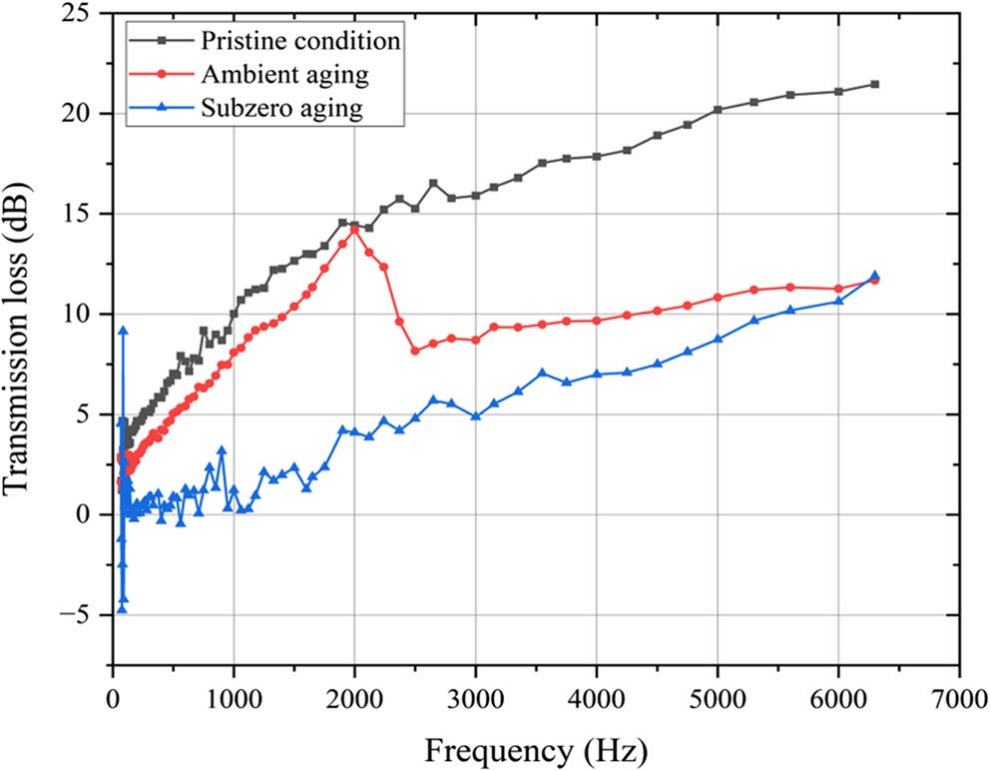
Transmission loss vs. frequency for pristine and aged conditions.

Sound absorption is a phenomenon in which a portion of incident sound energy is converted into thermal or mechanical energy within a material of finite thickness. This process, also termed as acoustic absorption, is instrumental in sound attenuation. Quantified in decibels, it represents the reduction in sound intensity as the wave traverses through the material. Figure 23 graphically illustrates the relationship between transmission loss and frequency for the three experimental conditions as determined through impedance tube testing.

In the pristine condition, the sound transmission loss (STL) at 6300 Hz was found to be 21.458 dB. The STL curve is characterised by a high number of peaks and dips, particularly between 0 and 1000 Hz and 1800–3000 Hz. Basalt fibers provide high strength, good thermal stability and good resistance to chemical and environmental degradation. Epoxy resin, known for its strong adhesive properties and mechanical strength, contributes to the composite’s durability and rigidity. Together, these components form a composite with exceptional sound attenuation characteristics. The various peaks show effective sound attenuation at specific frequencies, but the dips indicate frequencies where the composites permit more sound to travel through. STL gradually increases after 3000 Hz, with a tiny dip between 4000 and 5000 Hz, indicating the material’s strong performance at these frequencies.

Under ambient conditions, the sound transmission loss (STL) at 6300 Hz for the basalt fiber and epoxy resin composite was measured at 11.6 dB. The STL curve shows fewer peaks and dips compared to the pristine and subzero conditions. Initially, an optimal STL of 14.2 dB was observed at 2000 Hz, followed by a significant dip and a gradual increase, reaching an STL of 11.6 dB at 6300 Hz. Basalt fiber-reinforced composites perform satisfactorily under normal climatic conditions. However, exposure to fluctuating temperatures, humidity, and atmospheric agents can induce microstructural changes within the fiber’s porous architecture. This reduces the material’s effectiveness in attenuating sound at certain frequencies, resulting in pronounced attenuation at frequencies above 2000 Hz. The fewer peaks and dips indicate a more stable but less effective sound attenuation performance across a broad range of frequencies.

Under subzero temperatures, the sound transmission loss (STL) at 6300 Hz for the basalt fiber and epoxy resin composite was observed to be 12.5 dB. The STL curve shows many peaks and dips, especially between 1000 and 2000 Hz, with several peaks extending up to 4000 Hz before stabilizing and gradually increasing to 12.5 dB at 6300 Hz. Low temperatures can affect the flexibility and density of the epoxy resin, making it more brittle and potentially reducing its damping properties. While basalt fibers may retain their strength at low temperatures, the overall composite can experience variations in its acoustic properties. The subzero conditions demonstrate that the materials remain highly responsive to sound frequencies within the 1000–4000 Hz range. These composites can provide effective acoustic performance in applications such as partitions, flooring, hull insulation, and interior panels.

## Conclusion

This study comprehensively examined the effects of subzero and ambient aging on the mechanical and vibrational characteristics of quasi-isotropic basalt fiber-reinforced epoxy composites. The results are summarised as follows,

- Ambient aged specimens exhibited the highest moisture content of 8.66%, while the subzero aged specimens recorded 5.44% of moisture at saturation.
- The tensile behaviour of the composite revealed ultimate tensile strength of 236.7 MPa for pristine conditions, 179.48 MPa for ambient-aged, and 205.02 MPa for subzero-aged specimens. SEM analysis of the tensile fracture surfaces identified fiber breakage, debonding, degradation of the matrix, and weak interfacial bonding as primary damage mechanisms in aged specimens.
- The maximum degradation in flexural strength was observed in ambient aged specimens followed by subzero aged specimens with retention rates of 68.3% and 37.6%, respectively compared to pristine specimens. Flexural fracture surfaces across all conditions showed a wavy pattern of compression buckling and matrix fragmentation, which was more pronounced in aged specimens.
- Short beam shear strength (SBS) exhibited significant degradation with values of 7.52 MPa for ambient-aged, and 8.35 MPa for subzero-aged specimens compared to 10.17 MPa for pristine specimens. SEM images revealed the presence of wavy matrix crack patterns, matrix fragmentation, and compression buckling in the aged specimens.
- Pristine samples demonstrated the maximum impact strength of 114.08 kJ/m^2^. The impact strength retention for the aged samples, was found to be 91.43% for the subzero samples and 80.6% for the ambient samples.
- Vibrational analysis indicated natural frequencies of 8.25 Hz for pristine, 6 Hz for ambient-aged, and 7 Hz for subzero-aged specimens. Sound absorption tests showed that pristine specimens had the highest transmission loss, while moisture-rich ambient-aged specimens exhibited the lowest.
- The study shows that both ambient and subzero aging significantly reduce the mechanical and vibrational properties of basalt fiber-reinforced epoxy composites, with subzero conditions being less damaging, highlighting the need for developing more durable composites to enhance performance in demanding engineering applications.
- The findings highlight the need for enhanced moisture resistance and durability in quasi-isotropic basalt fiber composites for long-term use in varying environmental conditions. Future research should explore improved matrix systems, fiber treatments, and aging scenarios like freeze-thaw cycles to address these challenges.

## Data Availability

The datasets generated during and/or analyzed during the current study are available from the corresponding author upon reasonable request.
